# The Importance of Integration of Stakeholder Views in Core Outcome Set Development: Otitis Media with Effusion in Children with Cleft Palate

**DOI:** 10.1371/journal.pone.0129514

**Published:** 2015-06-26

**Authors:** Nicola L. Harman, Iain A. Bruce, Jamie J. Kirkham, Stephanie Tierney, Peter Callery, Kevin O'Brien, Alex M. D. Bennett, Raouf Chorbachi, Per N. Hall, Anne Harding-Bell, Victoria H. Parfect, Nichola Rumsey, Debbie Sell, Ravi Sharma, Paula R. Williamson

**Affiliations:** 1 MRC North West Hub for Trials Methodology Research, Department of Biostatistics, University of Liverpool, Liverpool, United Kingdom; 2 Central Manchester University Hospitals NHS Foundation Trust, Royal Manchester Children’s Hospital, Manchester, United Kingdom; 3 Royal College of Nursing Research Institute, Warwick Medical School, University of Warwick, Warwick, United Kingdom; 4 School of Nursing, Midwifery and Social Work, Jean McFarlane Building, University of Manchester, Manchester, United Kingdom; 5 The Healing Foundation Cleft and Craniofacial Clinical Research Centre, School of Dentistry, University of Manchester, Manchester, United Kingdom; 6 NHS Lothian, ENT Department, Lauriston Place, Edinburgh, United Kingdom; 7 North Thames Cleft Service and the Department of Audiological Medicine/ Audiology/Cochlear implants. Great Ormond Street Hospital for Children, London, United Kingdom; 8 Cleft Net East, Cambridge University Hospital NHS Trust, Hills Road, Cambridge, United Kingdom; 9 Centre for Appearance Research, Department of Health & Social Sciences, University of the West of England, Bristol, United Kingdom; 10 North Thames Cleft Service, Speech and Language Therapy, Great Ormond Street Hospital for Children, London, United Kingdom; 11 North West, Isle of Man and North Wales Cleft Lip and Palate Network, Alder Hey Children’s NHS Foundation Trust, Liverpool, United Kingdom; Medical University of South Carolina, UNITED STATES

## Abstract

**Background:**

Approximately 75% of children with cleft palate (CP) have Otitis Media with Effusion (OME) histories. Evidence for the effective management of OME in these children is lacking. The inconsistency in outcome measurement in previous studies has led to a call for the development of a Core Outcome Set (COS). Despite the increase in the number of published COS, involvement of patients in the COS development process, and methods to integrate the views of patients and health professionals, to date have been limited.

**Methods and Findings:**

A list of outcomes measured in previous research was identified through reviewing the literature. Opinion on the importance of each of these outcomes was then sought from key stakeholders: Ear, Nose and Throat (ENT) surgeons, audiologists, cleft surgeons, speech and language therapists, specialist cleft nurses, psychologists, parents and children. The opinion of health professionals was sought in a three round Delphi survey where participants were asked to score each outcome using a bespoke online system. Parents and children were also asked to score outcomes in a survey and provided an in-depth insight into having OME through semi-structured interviews. The results of the Delphi survey, interviews and parent/patient survey were brought together in a final consensus meeting with representation from all stakeholders. A final set of eleven outcomes reached the definition of “consensus in” to form the recommended COS: hearing; chronic otitis media (COM); OME; receptive language skills; speech development; psycho social development; acute otitis media (AOM); cholesteatoma; side effects of treatment; listening skills; otalgia.

**Conclusions:**

We have produced a recommendation about the outcomes that should be measured, as a minimum, in studies of the management of OME in children with CP. The development process included input from key stakeholders and used novel methodology to integrate the opinion of healthcare professionals, parents and children.

## Introduction

Cleft lip and palate has an incidence of around 1 in 700 individuals making it one of the most common congenital malformations worldwide [1]. In children with cleft palate (CP) there is a tendency towards Eustachian tube dysfunction, which can contribute to the development and persistence of negative middle ear pressure and the accumulation of mucoid or serous fluid within the middle ear space (Otitis Media with Effusion (OME), glue ear)[2,3]. The tendency to develop OME is greater, and persists for longer, in children with CP. Consequently, approximately 75% of children with CP will have a history of non-trivial OME [1,4].

The consequences of persistent OME can include increased tendency to develop ear infections (acute otitis media, AOM), long-term middle ear problems (chronic otitis media, COM) and hearing loss, which can have a negative impact on speech and language development, communication, behavior and educational attainment. There are several approaches to the management of OME in children with clefts and they include watchful waiting, the provision of hearing aids and the insertion of ventilation tubes. However, the evidence underpinning these strategies is not clear, particularly for children with CP [5].

The MOMENT study (Management of Otitis Media with Effusion in childreN with cleft palaTe) was a feasibility study designed in response to a commissioned call from the National Institute of Health Research, Health Technology Assessment Programme to answer the question “What is the most appropriate way to manage otitis media with effusion in children with cleft palate?”. There is currently no Core Outcome Set (COS) for clinical trials of the management of OME in children with CP [6]. Therefore, one objective of the study was the development of a COS relevant to the treatment of OME in children with CP.

A Core Outcome Set represent the minimum that should be measured and reported in effectiveness trials in a particular condition [7]. The use of a minimum set of core outcomes aims to increase consistency of reporting in clinical trials. This has been demonstrated for trials in rheumatological conditions with an increase in the consistency of outcome reporting following the publication of a COS [8]. A systematic review directed at the early routine insertion of ventilation tubes for the management of OME in children with CP identified a variety of primary and secondary outcomes together with inconsistency in the method of measurement [9]. The use of an agreed set of core outcomes, measured and reported in all randomized controlled trials (RCTs) of treatments for OME in children with CP, could overcome well documented issues of heterogeneity and outcome reporting bias (ORB) [10–12], whilst at the same time increasing the potential for meta-analyses of key outcomes in this area.

Specific objectives of the COS development in the MOMENT study were: to identify outcomes that had been previously reported in studies of the treatment of OME; to prioritise outcomes from the perspective of health professionals; to prioritise outcomes from the perspective of patients who can express their views, and parents; and to integrate the opinions of patients, parents and health professionals into a combined COS.

### Limitations of previous methods for COS development

Less than a fifth of previous COS studies have involved public representatives, and the majority of those included only a handful of patients [11]. Despite evidence that patients may hold different views from health professionals, and a recommendation to include patients in the process [9], it is unclear whether different stakeholder group views were transparent to those involved [11]. This current study implemented a novel design for COS development, including a new method proposed by children and young people to elicit opinion from children. We investigated the influence of the method of stakeholder feedback on subsequent opinion. We report the results from this work here.

## Methods

**Fig 1:**
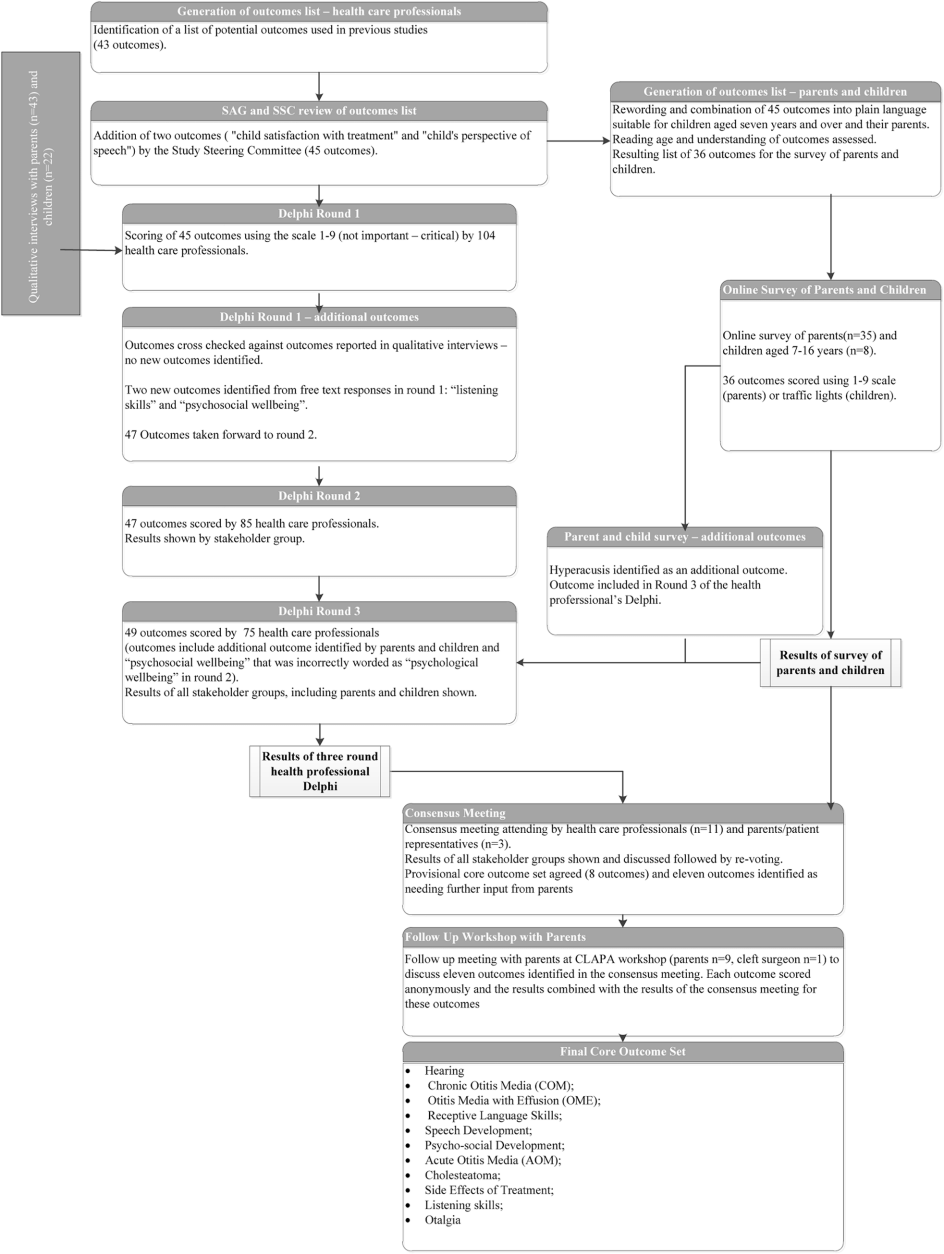
Overview of the COS development process.

The study protocol for this work, including search strategy and inclusion criteria for the systematic review, has been previously published ***[13]***. Methods used for the systematic review, health professional Delphi survey, semi-structured interviews and final study consensus meeting are described briefly below. An online survey of parents and children is described more fully. An overview of the COS development process is provided in Fig 1.

### Systematic Review and generation of the list of outcomes

A list of outcomes previously reported in studies of the treatment of OME was generated by updating a 2009 systematic review [9] using the same search strategy. The review of papers was completed by two authors independently (NLH and IAB) and a list of all outcomes measured in identified papers generated (detailed information is given in the protocol) [13]. This list of outcomes was further refined to standardize the name given to each of the outcomes. All outcomes and domains were discussed with members of the Study Advisory Group (SAG) prior to being finalised. Due to the varied health professional groups likely to be completing the Delphi an ‘outcome tip’, further describing the outcome, was also written for each outcome and reviewed by the SAG. The SAG comprised of Speech and Language Therapists (n = 2), Cleft Surgeons (n = 1), ENT Surgeons (n = 2), Audiologists (n = 2) and Clinical Psychologists (n = 1). A patient representative was approached during the study and accepted membership of the SAG but then, due to unforeseen personal circumstances, needed to withdraw from membership. prior to attending an SAG meeting. The SAG and also the Study Steering Committee (SSC, comprising a trial methodologist, patient representative, health economist and a paediatric otorhinolaryngologist) were also given the opportunity to add outcomes to the list that they considered important.

### Health Professional Delphi Survey

The opinion of health professionals was sought through a three round Delphi survey delivered using a bespoke online system[13]. Health professionals were eligible to participate in the Delphi survey if they were affiliated to a UK Cleft Centre and were a cleft surgeon, ENT surgeon, audiologist, cleft nurse specialist, clinical psychologist or speech and language therapist. Potentially eligible health professionals were identified through contact with clinical leads at each of the 15 UK centres who provided a list of current members of their cleft team and their clinical role (S3 Table).

Prior to completion of round 1 it was agreed that for the feedback of results in round 2 to be presented by stakeholder groups, approximately 10 participants per stakeholder group would be required for the presentation of results to be meaningful. In the second round results were presented by health professional stakeholder group, with an individual seeing only the aggregated results from their particular group together with a reminder of their own round 1 score. In the third and final round the results of all stakeholder groups, including parents and children, presented separately, were shown to each participant together with a reminder of their round 2 score.

In each round of the Delphi survey, health professionals were asked to score a list of outcomes using the Grading of Recommendations, Assessment, Development and Evaluations scale of 1 to 9, with 1 to 3 labelled ‘not important’, 4 to 6 labelled ‘important but not critical’ and 7 to 9 labelled ‘critical’ [14] (S1 Fig). Consensus on outcomes for inclusion in the COS was determined using a pre-defined definition of consensus (S1 Table).

### Opinions of patients and parents

The views of parents of children with a CP aged 0–11 years, and children with a CP aged 6–11 years, were explored in semi-structured interviews conducted by one of the authors (ST), who does not have a clinical background and was not known to parents or children prior to the interview. A purposive sample was recruited to provide maximum variation in terms of a child’s age and gender and type of treatment experienced for OME. Participants were recruited from two cleft centres in the UK with contrasting approaches to audiology care, one a centralized service, the other distributed across a hub and spoke” model. Interviews were audio-recorded, transcribed verbatim, and then Framework analysis [14] was used to manage and interpret data. Discussion around outcomes took place throughout interviews. However, there was a specific section of the topic guide used within interviews that focused on capturing data on this issue. Results of interviews relating to experiences of OME are reported elsewhere [15,16].

Semi-structured interviews with parents and children gave in-depth information on outcomes of importance for these groups. Interviews did not include a discussion of the outcomes list generated through systematically reviewing the literature because we wanted participants to express their opinions, in their own words, of what they felt were important results or indicators of successful management of OME. In order to give parents and children the same opportunity to score outcomes, an online survey, similar to that completed by health professionals, was developed. This involved review and re-wording, using a plain language description, of each outcome scored by health care professionals. Each re-worded outcome was tested for readability using the NIACE SMOG calculator [17]. Understanding was explored with the Cleft Lip and Palate Association (CLAPA) children and young person’s council (CYPC) and a local CLAPA ‘Happy Faces’ group. The same outcome wording was used for all participants with the exception of minor changes such as “your/your child’s” to ensure appropriate context.

**Fig 2:**
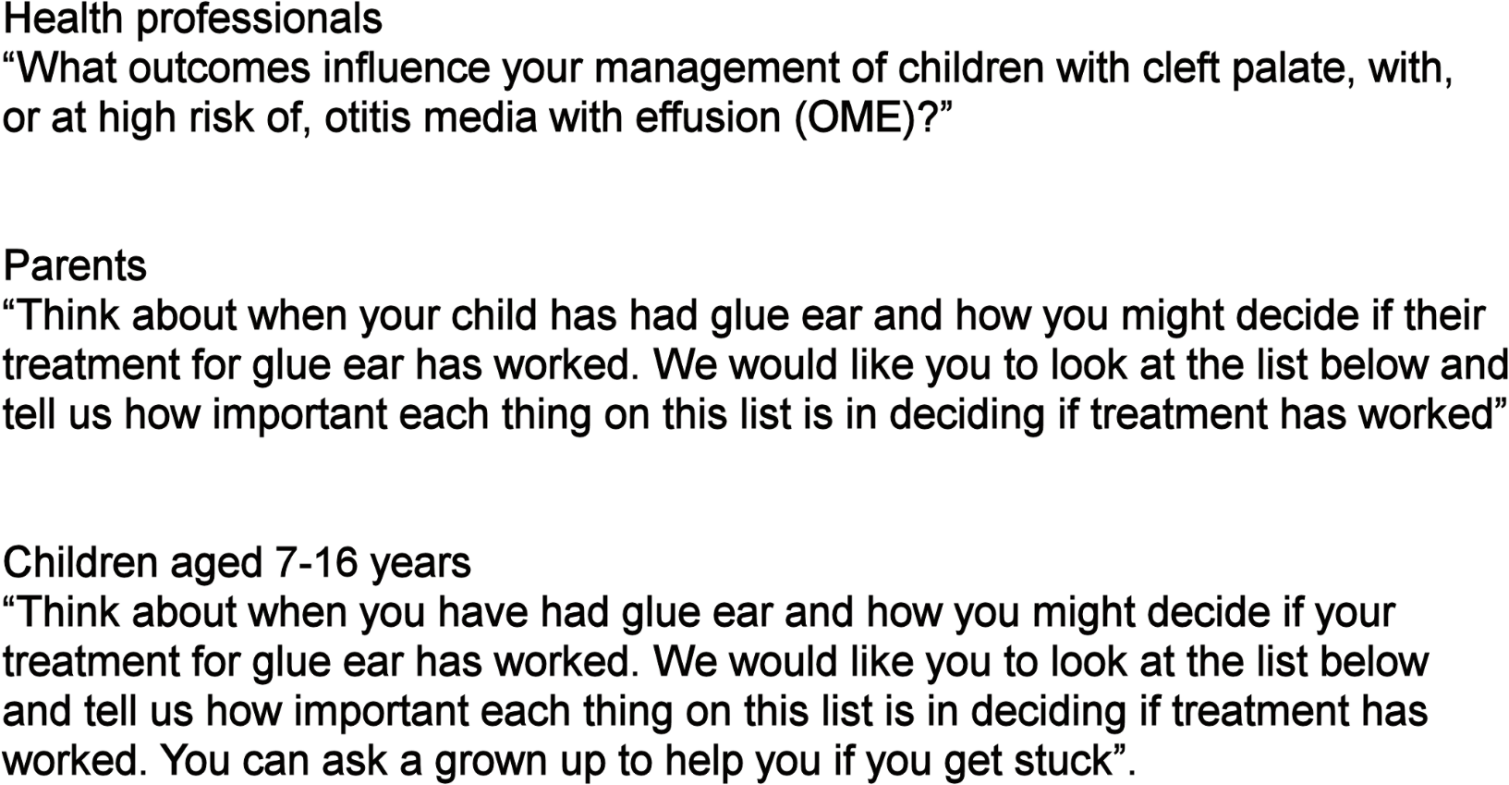
Initial question asked prior to scoring outcomes for parents, adults and children with cleft palate. The question asked of health professionals is included for comparison.

Parents and children aged 7–16 years were asked to consider the appropriate question, described in Fig 2, and to score each of the outcomes. The labels of the 1–9 scale were modified for parents whilst children, under the recommendation of the CYPC, scored each outcome using a traffic light system where the scores 1–3 were represented by a red box labelled as “not that important”, scores 4–6 as an amber box labelled as “important” and scores 7–9 as a green box labelled “really important” (S1 Fig). Both parents and children were provided with a free text box to add anything else that they considered relevant. The definition of consensus (S1 Table) was applied to the results from both parents and children.

Participants of the survey for parents and children were independent of those who completed a semi-structured interview and were identified using the CLAPA mailing list and social media pages with a potential reach of 4,710 and 9,564 respectively. An individual email was sent to all those on the CLAPA mailing list together with a reminder in their e-newsletter. A link was posted on the Facebook page which included the researcher’s name and photograph (NLH) and a link to the online survey. There is likely to be substantial overlap with membership of multiple Facebook pages and groups however, it was not possible to assess this.

### Final consensus meeting

The consensus meeting brought together all sources of information. The results from the Delphi survey of health professionals and the survey of patients and parents were presented along with the opinions from 43 parents and 37 children who took part in a semi structured interviews. An invitation to attend was sent to: health professionals who had completed all rounds of the online Delphi survey and expressed an interest in attending future meetings; all parents who had completed an online survey and expressed an interest and provided contact details to be informed about future meetings; parents who had taken part in a semi-structured interview whose contact details were still valid; CLAPA members in the North West who subscribed to the CLAPA mailing list. The format of the consensus meeting comprised a short study overview, a summary of results from the semi-structured interviews and a review of each outcome on the scored list in turn, including presentation of how each stakeholder group had scored the outcome, and the number of stakeholder groups who achieved consensus. Discussion of each outcome was followed by anonymous electronic scoring by those at the consensus meeting. A written report of the final meeting was circulated to participants for comment.

### Ethics statement

Ethical approval was received from the National Research Ethics Service North West – Greater Manchester East Research Ethics Committee (Reference 11/NW/0586) for the completion of semi-structured interviews and invitation of interviewees to the final consensus meeting. Written consent was sought for participation in semi-structured interviews, with written proxy consent sought from parents/guardians for their child’s participation. Written assent was also sought from children aged 6 years and older. Attendance at the consensus meeting was considered to be implied consent for participation with no written consent provided, this process was approved by the Research Ethics Committee. Advice was sought from the National Research Ethics Service who did not consider that ethical approval was required for an online survey of parental and child opinion. However, full information about initial the study was given in the initial pages of the online survey and survey completion considered to imply consent.

## Results

### Systematic review

The search retrieved 85 potentially eligible studies with an additional 42 identified from other sources, after screening titles and abstracts, all but nine studies were deemed to be irrelevant (S2 Fig). After further analysis of the full texts one further study was excluded as it was undertaken to determine the frequency that children with CP pass their new born hearing test [18]. Two non-English papers were identified [19,20], for these the abstract and the review by Ponduri *et al* were used to assess eligibility and extract outcomes. A total of 49 studies were included: eight studies of children with CP [3,21–27]; seventeen studies [19,20,28–42] identified in the previous review [9] and 24 studies [43–66] identified from six Cochrane systematic reviews relating to OME [67–72].

### Generation of an outcome list

Each outcome measured was listed by study. Only individual outcomes were included, for example, where an outcome was measured using different methods this was counted as one outcome but the methods of measurement noted (S2 Table).

The number of outcomes measured in an individual study varied with a median of 6 outcomes (range 1–14 outcomes) per paper. Outcomes related to resource use were considered to be outside the scope of the COS. Consequently, the outcomes, “necessity to visit doctor” and “level of speech therapy support required” were not considered in the list of outcomes. The final list of outcomes used in round 1 of the Health Professionals Delphi comprised 45 individual outcomes (43 identified from the systematic review and two added by the Study Steering Committee) grouped under 14 domains (Table 1). The list of outcomes scored by parents and children included the combination of some outcomes, for example those that related to specific clinical observations, so that a total of 36 outcomes were scored (Table 1).

**Table 1:** Outcomes scored by health professionals, parents and children.

Original Outcome	Outcome Domain	7-10yrs	11–16 yrs	Parents
Internalising Behaviour	Things about behaviour/ Things about your child's behaviour/Things about behaviour	How lonely you feel, feeling like an outsider	How lonely you feel, feeling like an outsider	How lonely your child feels, feeling like an outsider
Externalising Behaviour	Things about behaviour/ Things about your child's behaviour/Things about behaviour	How angry you are towards others	How angry you are towards others	How angry your child is towards others
Atelectasis, persistent tympanic membrane retraction, tympanosclerosis	Things about having problems with your ears for a long time/ Things about your child having problems with their ears for a long time	Not having problems inside your ear caused by having lots of ear infections over a long time (more than 3 months)	Not having problems inside your ear caused by having lots of ear infections over a long time (more than 3 months)	Your child not having problems inside their ear caused by having lots of ear infections over a long time (more than 3 months)
Cholesteatoma	Things about having problems with your ears for a long time/ Things about your child having problems with their ears for a long time	Not having problems inside your ear caused by bad skin growing behind your ear drum.	Not having problems inside your ear caused by bad skin growing behind your ear drum.	Your child not having problems inside their ear caused by bad skin growing behind your ear drum.
Chronic Otitis Media	Things about having problems with your ears for a long time/ Things about your child having problems with their ears for a long time	Not having problems inside your ear caused by having glue ear for a long time (more than 3 months)	Not having problems inside your ear caused by having glue ear for a long time (more than 3 months)	Your child not having problems inside their ear caused by having glue ear for a long time (more than 3 months)
Persistent tympanic membrane perforation	Things about having problems with your ears for a long time/ Things about your child having problems with their ears for a long time	Not having problems inside your ear caused by having a hole in your ear drum for a long time (more than 3 months	Not having problems inside your ear caused by having a hole in your ear drum for a long time (more than 3 months	Your child not having problems inside their ear caused by having a hole in your ear drum for a long time (more than 3 months
Academic achievement, cognitive development,developmental progress, intelligence, literacy, phonological memory	Things about school and making friends	How well you are doing at school	How well you are doing at school or college	How well your child is doing at school or college
Psycho social development	Things about school and making friends	How well you are learning to make friends and speak to new people	How well you are learning to make friends and speak to new people	How well your child is learning make friends and speak to new people
Hearing	Things about how your ear feels and works/Things about how your child's ear feels and works	How well you can hear	How well you can hear	How well your child can hear
Otalgia	Things about how your ear feels and works/Things about how your child's ear feels and works	How painful your ear is	How painful your ear is	How painful your child's ear is
Otorrhoea	Things about how your ear feels and works/Things about how your child's ear feels and works	Not having infected liquid leaking out of your ear	Not having pus (infected liquid) leaking out of your ear	Your child not having pus (infected liquid) leaking out of their ear
Tinnitus	Things about how your ear feels and works/Things about how your child's ear feels and works	How much you hear buzzing or ringing noises	How much you hear buzzing or ringing noises	How much your child hears buzzing or ringing noises
Vertigo	Things about how your ear feels and works/Things about how your child's ear feels and works	How dizzy you feel	How dizzy you feel	How dizzy your child feels
Eustachian tube function	Things about how the middle part of your ear works/Things about how the middle part of your child's ear works	How well a special tube in your ear works. If this tube doesn’t work properly you might hear popping and crackling noises.	How well a special tube in your ear works. If this tube doesn’t work properly you might hear popping and crackling noises.	How well a special tube in your child's ear works. If this tube doesn't work properly your might hear popping and crackling noises.
Stapedial reflex	Things about how the middle part of your ear works/Things about how the middle part of your child's ear works	How well your ear works when it hears a loud noise	How well your ear works when it hears a loud noise	How well your child's ear works when it hears a loud noise
Nasal obstruction	Things about how your nose feels/Things about how your child's nose feels	How well you can breathe through your nose	How well you can breathe through your nose	How well your child can breathe through their nose
Rhinitis	Things about how your nose feels/Things about how your child's nose feels	How much your nose feels runny or stuffy	How much your nose feels runny or stuffy	How much your child's nose feels runny or stuffy
Acute otitis media (AOM)	Things about glue ear and ear infections	Not having ear infections	Not having ear infections	Your child not having ear infections
Otitis media with effusion (OME)	Things about glue ear and ear infections	Not having glue ear and being able to hear better	Not having glue ear and being able to hear better	Your child not having glue ear
Temporary tympanic membrane perforation	Things about glue ear and ear infections	Not having a hole in your eardrum that only lasts for a few weeks	Not having a hole in your eardrum that lasts for a few weeks	Your child not having a hole in their eardrum that lasts for a few weeks
Consonant production, consonant production—cleft related speech patterns, expressive language skills	Things about talking	Being able to say all your words clearly and grownups and children understanding what you say	Being able to say all your words clearly and grownups and children understanding what you say	Your child being able to say all their words clearly so that adults and other children can understand what they said
Parent's perspective of speech	Things about talking	How much you talk like someone without a cleft palate	How much you talk like someone without a cleft palate	How much your child talks like someone without a cleft palate
Receptive language skills	Things about talking	Being able to listen and understand what other people say	Being able to listen and understand what other people say	Your child being able to listen and understand what other people say
Speech development	Things about talking	How well your parents think you are speaking	How well your parents think you are speaking	How well you think your child is speaking
Speech intelligibility	Things about talking	Speaking as well as other children the same age as you	Speaking as well as other children the same age as you	Your child speaking as well as other children who are the same age
Speech signs of velopharyngeal insufficiency	Things about talking	Your speech not sounding different to other children	Your speech not sounding different to other children	Your child's speech not sounding different to other children
Early extrusion or blockage of ventilation tubes	Things about grommets	How often your grommets/ventilation tubes fall out or don't work	How often your grommets/ventilation tubes fall out or don't work	How often your child's grommets/ventilation tubes fall out or don't work
Necessity to remove ventilation tubes	Things about grommets	Not needing another operation to take grommets/ventilation tubes out	Not needing another operation to take grommets/ventilation tubes out	Your child not needing another operation to take grommets/ventilation tubes out
Original Outcome	Outcome Domain 7–16 year old and adults/parents	Outcome 7-10yrs	Outcome 11–16 yrs	Outcome Parents
Requirement for repeated ventilation tubes	Things about grommets	Not needing another operation to have new grommets/ventilation tubes because the old ones fell out.	Not needing another operation to have new grommets/ventilation tubes because the old ones fell out.	Your child not needing another operation to have new grommets/ventilation tubes because the old ones fell out.
Child stress	Things about how you or your parents feel/ Things about how you or your child feels	How often you feel upset or angry	How often you feel upset or angry	How often your child feels tense or upset
Parental stress	Things about how you or your parents feel/ Things about how you or your child feels	How often your parents feel upset or angry	How often your parents feel upset or angry	How often you feel tense or upset
Parental satisfaction with treatment	Things about how well your child's treatment has worked	How well your parents think that hearing aids or grommets have improved your hearing	How well your parents think that hearing aids or grommets have improved your hearing	How well you think that hearing aids or grommets have improved your child's hearing
Side effects of Treatment	Things about problems caused by treatment/Things about problems caused by your child's treatment	Not having problems, that can sometimes happen, that are caused by a treatment you have for glue ear	Not having problems, that can sometimes happen, that are caused by a treatment you have for glue ear	Your child not having problems, that can sometimes happen, that are caused by a treatment they have for glue ear
Upper Respiratory Tract Infection	Things about infections in the ear, nose or mouth	Not having infections in your ear, nose or mouth	Not having infections in your ear, nose or mouth	Your child not having infections in their ear, nose or throat
Child's satisfaction with treatment	Other things	How much you think treatment has made you better	How much you think treatment has made you better	How much your child thinks that treatment has made them better
Child's perspective of speech	Other things	How normal you think you sound when you are talking	How normal you think you sound when you are talking	How normal your child thinks they sound when they are talking
Psychological wellbeing	Not scored	Not scored	Not scored	Not scored
Listening skills	Not scored	Not scored	Not scored	Not scored
Psychosocial wellbeing	Not scored	Not scored	Not scored	Not scored
Hyperacusis	Not scored	Not scored	Not scored	Not scored

### Identification of outcomes of importance to parents and children with CP

Semi-structured interviews were completed with 43 parents of 37 children, and 22 children, according to the sampling matrix described in the trial protocol [13]. Interviews with parents lasted, on average, 40 minutes, whilst those with children took, on average, 20 minutes. Parent and child responses to specific questions about outcomes were cross checked against the outcomes list generated from the systematic review of the literature and, as the semi-structured interviews were in parallel to the first round of the health professionals Delphi, were also checked against any free text responses provided by health professionals in round 1. Two investigators (IAB and NLH) mapped each outcome from the interviews against the list of outcomes after round 1. No new outcomes were identified.

### Online survey of parents and children

Two hundred and ninety three people accessed the online survey and 253 answered the initial question regarding eligibility. Of the 235 eligible only 51 (22%) completed the survey. Responses were received from 35 parents, eight adults and eight children. Of the eight children, four were in the 7–10 years age group and four aged 11–16 years.

The results were reviewed against the definition of consensus agreed prior to the start of the study [13]. Using this definition, parents and children had reached “consensus in” for 35 and 11 outcomes respectively (Table 2).

**Table 2:** Summary of all groups reaching consensus for individual outcomes scored in the health professional Delphi survey and online survey for parents and children.

Outcome	Round 3 and survey of parents and children with CP							
	Cleft Surgeon	ENT Surgeon	Specialist Cleft Nurse	Speech and Language Therapist	Psychologist	Audiologist	Parent	Child
Internalising Behaviour			*		*			
Externalising Behaviour			*		*			
Atelectasis	*		*			*	*	*
Cholesteatoma	*	*	*	*		*		
Chronic Otitis Media	*	*	*	*		*	*	*
Persistent tympanic membrane perforation	*		*					*
Persistent tympanic membrane retraction	*		*				*	*
Tympanosclerosis			*				*	*
Academic achievement			*				*	
Cognitive development			*	*	*		*	
Developmental progress			*	*	*		*	
Intelligence							*	
Literacy			*				*	
Phonological memory				*			*	
Psycho social development			*	*	*		*	*
Hearing	*	*	*	*	*	*	*	*
Otalgia	*		*				*	
Otorrhoea	*	*				*		
Tinnitus			*				*	
Vertigo			*				*	
Eustachian tube function	*		*				*	
Stapedial reflex			*				*	
Nasal obstruction								
Rhinitis								
Acute otitis media (AOM)	*		*	*		*	*	
Otitis media with effusion (OME)	*	*	*	*		*	*	*
Temporary tympanic membrane perforation			*					
Consonant production	*		*	*		*	*	
Consonant production—cleft related speech patterns	*		*	*			*	
Expressive language skills			*	*			*	
Parent's perspective of speech				*			*	
Receptive language skills	*		*	*		*	*	*
Speech development	*	*	*	*		*	*	
Speech intelligibility	*	*	*	*		*		
Speech signs of velopharyngeal insufficiency			*	*			*	
Early extrusion or blockage of ventilation tubes			*			*	*	
Necessity to remove ventilation tubes	*		*			*	*	*
Requirement for repeated ventilation tubes	*		*			*	*	*
Child stress			*		*		*	
Parental stress			*		*			
Parental satisfaction with treatment		*	*		*	*	*	
Side effects of treatment		*	*			*	*	
Upper respiratory tract infection							*	
Child's satisfaction with treatment			*	*	*	*	*	
Child's perspective of speech			*	*	*	*	*	
Psychological wellbeing^†^			*	*	*		/	/
Listening skills^†^	*		*	*		*	/	/
Psychosocial wellbeing^†^	*		*	*	*		/	/
Hyperacusis^†^							/	/

† Not scored by parents or children

### Identification of Outcomes of Importance to Health Professionals

#### Round 1 –Health Professionals

The overall response rate per round is given in S4 Table. The number of outcomes reaching consensus within each stakeholder group in each round is shown in Table 3.

**Table 3:** Number of outcomes achieving consensus.

	Number of outcomes reaching consensus in round 1 and staying in consensus throughout	Additional outcomes achieving consensus in round 2 compared to round 1	Additional outcomes achieving consensus in round 3 compared to round 2
Cleft Surgeon	14	10	0^†^
ENT Surgeon	4	0	3
Specialist Cleft Nurse	32	3	4
Speech and Language Therapist	13	10	0*
Psychologist	7	4	1
Audiologist	6	13	0

^†^ six fewer outcomes achieved consensus in round 3 compared to round 2

* one less outcome achieved consensus in round 3 compared to round 2.

The 18 free text responses provided by health professionals in round 1 were reviewed by the SMG and SAG. Eight responses represented a comment and two relating to the use of hearing aids were considered outside the scope of the study. The remaining eight described potential outcomes of which two (listening skills and psychosocial wellbeing) were not already represented and therefore were taken forward to round 2. Thus in round two health professionals scored 47 outcomes.

#### Round 2 –Health Professionals

Of the 104 participants who completed round 1 only 99 were eligible to participate in round 2: three clinical geneticists were excluded from further rounds as they were not involved in the management of OME and two participants were on maternity leave at the time of round 2 and therefore would not be able to participate further. A total of 85 responses were received in round 2 (86% of those completing round 1 and eligible for round 2). Participants were shown their own score from round 1 alongside the percentage of participants giving each score from their own stakeholder group. They were informed that they could change their score or keep it the same as their score in round 1. The median percentage of scores changed between round 1 and 2 was 18% (range 0–100%). One participant changed 100% of their score in round 1 whilst six participants (7%) made no changes to their scores.

#### Round 3- Health Professionals

After round 2, four participants left the cleft service and so were no longer eligible to participate in round 3. A total of 73 responses were received (90% of those completing round 2 and eligible to complete round 3). All sites were represented in the responses to round 3 with a variable representation of sites and health professional stakeholder groups within site (S4 Table). In round 3, 49 outcomes were scored. One additional outcome “hyperacusis” (sensitivity to loud noises) was identified from free text responses to the parent/child survey. A typing error in the entry of outcomes onto the online system in round 2 had led to “psychosocial wellbeing” being listed as “psychological wellbeing” which is considered to be a different outcome. Therefore in round 3 this was clarified and participants asked to score “psychosocial wellbeing” as well as re-score “psychological wellbeing”. This time, participants were shown their own score in round 2, together with the scores for each of the stakeholder groups including parents and children. The median percentage of scores changed between round 2 and 3 was 21% (range 0–83%). Six participants (8%) made no changes to their scores and no participants changed all scores.

#### Consensus Matrix

The scores in round 3 were compared against the definition of consensus to determine which stakeholder groups had reached the definition of “consensus in”. After round 3 all eight stakeholder groups (health professionals plus parents and children) had reached “consensus in” for one outcome “hearing”. Results for all outcomes are given in Table 3.

#### Attrition bias between rounds

To identify whether attrition in round 2 would introduce bias, the average score across outcomes from round 1 was calculated for each participant and then compared for those completing both rounds (n = 85) versus those completing round 1 only (n = 14). Likewise in round 3, scores were compared for those completing both rounds 2 and 3 (n = 73) versus those completing round 2 only (n = 8). The results of those who did not complete round 2 or round 3 did not represent extreme views suggesting that bias had not been introduced through attrition between rounds (S3 and S4 Figs).

#### Variability in outcomes achieving consensus between rounds

Consensus was reached on additional outcomes within all health professional groups when shown results from either their own stakeholder group, or all groups plus those from parents and children with CP, or both (Table 3). This suggests the Delphi, as opposed to a one-off survey, was a useful exercise.

### Consensus Meeting

Twenty five participants attended the consensus meeting of whom 14 were eligible to vote. All stakeholder groups with the exception of clinical psychologists were represented (S6 Table).

Outcomes were discussed in the order of the number of stakeholder groups achieving consensus. Each outcome was categorized based on the following: 1 Discussed and voted (19 outcomes); 2 Discussed and agreed to combine with another outcome and to be considered as part of the “how” an outcome is measured (14 outcomes); 3 Discussed and agreed that further discussion with parents was needed (seven outcomes); 4 Agreed not to discuss further or vote—not in the COS (nine outcomes). A full breakdown of outcomes discussed at the consensus meeting is provided in S5 Table.

The evidence from the health professional Delphi, the parent and child survey and discussion at the consensus meeting followed by voting, were integrated and a COS proposed. This was then further discussed and approved at a follow up meeting of the SAG.

The consensus meeting followed by discussion with the SAG identified 11 outcomes that required further discussion with parents. For example, at the consensus meeting the outcome “listening skills” reached consensus for inclusion in the COS. This outcome was added by health care professionals as part of their Delphi and was not scored by parents or children. Consequently “listening skills” was considered to require further discussion with parents. Outcomes identified at the consensus meeting as requiring additional input, were discussed with parents at a follow up meeting held as a parallel workshop at the CLAPA annual conference, October 2014. Nine parents and one cleft surgeon took part in the workshop. The session included discussion and, if needed, further explanation of each outcome. Each outcome was scored anonymously using an electronic scoring system for immediate feedback. The scores from the workshop were combined with the scores from the consensus meeting and the definition of consensus applied. Following voting, “listening skills” was confirmed for inclusion in the COS. Two additional outcomes, “cholesteatoma” and “otalgia”, also reached consensus in. All outcomes meeting the definition of consensus and included in the recommended COS are shown in Table 4.

**Table 4:** Recommended core outcome set.

Outcome	Number of stakeholder groups scoring as “consensus IN”	Percentage scoring 7–9 at meeting	Percentage scoring 1–3 at meeting
Hearing	8	100%	0%
Chronic Otitis Media	7	100%	0%
Otitis media with effusion (OME)	7	93%	7%
Receptive language skills	6	100%	0%
Speech development	6	93%	7%
Psycho social development	5	71%	7%
Acute otitis media (AOM)	5	78%	7%
Cholesteatoma	5	71% ^†^	0%^†^
Side effects of treatment	4	100%	0%
Listening skills	4	91%^†^	0%^†^
Otalgia	3	82%^†^	14%^†^

^†^ - includes scores from follow up meeting with parents

## Discussion

This work has produced a consensus recommendation about what outcomes should be measured in studies related to the management of OME in children with CP and was undertaken as a component of the MOMENT study which investigated the feasibility of conducting an RCT in this area (Health Technology Assessment reference 09/167/02). The recommended COS includes: the presence of specific otological conditions (COM, OME and AOM); the potential physical or functional sequelae of these specific otological conditions (hearing loss, cholesteatoma, listening skills, psychosocial development, hearing, receptive language skills and speech development); and the potential negative consequences of treatment (side effects of treatment). The outcome “side effects of treatment” included in the COS will be dependent on the interventions/treatments that are being compared in a particular study

A recent systematic review of studies describing COS development demonstrated variability in stakeholder involvement with only 18% of studies including public representatives in the process [6]. The opinions of parents and children about the treatment of OME for children with CP are essential because this group will experience both the benefits and adverse effects of treatments, and be involved in decision-making about treatment. Importantly, the development of the COS in the MOMENT study has considered the opinion of patients and parents to ensure that outcomes regarded as most important, and included in the COS, are relevant to this stakeholder group.

We have demonstrated that clinical outcomes can be translated into plain language and that both parents and children are able to score these outcomes in an online survey. In this particular study, children were more discerning than their parents when considering which outcomes are most important to them with 11 outcomes reaching the definition of “consensus in”.

The use of an online survey of parents and children has allowed a broader range of outcomes to be considered than if interviews alone were used. Time constraints of the present study meant that only a one off survey was possible. However, future COS development should consider multiple rounds completed by patients/parents stakeholders in which the responses from health professionals can also be taken into account. Certainly the multiple rounds completed by health professionals resulted in changes being made to scores indicating that the responses of peers, parents and children and other health professional groups had an impact on the perceived importance of outcomes.

### Strengths and limitations

We have used an efficient online system to deliver a multiple round Delphi to health care professionals that allowed automated collating of scores and feedback in each round together with automated email alerts to promote completion. We have shown that individuals do reflect on, and are influenced by, other groups' opinions about the importance of outcomes. This online system was also successfully modified for use by parents and children in a one off survey.

Both clinical and patient stakeholders were engaged, with the response rate of health professionals similar to that reported in other Delphi surveys [73–75]. Notably the attrition rate was low with those taking part in round 1 likely to complete all rounds; as a result, no attrition bias was introduced. Whilst clinical stakeholder representation was good the number of parents and children completing the online survey and attending the face to face consensus meeting was lower than expected. In the present study it was not possible to ascertain whether the length of the survey for parents and children, the method of delivery or indeed the importance of the research question, due to perceived impact of OME, contributed to the low response rate. It is possible that those individuals completing the online survey are not representative of the wider group in terms of their views about important outcomes. Thirty seven parents were interviewed and these considered fewer outcomes to be critically important compared to parents who completed the online survey. However, with the exception of “externalising behaviour”, mentioned by one parent but not as the most important outcome, the opinions of those parents who were interviewed were comparable with the opinions expressed by parents completing the online survey. Research is needed on how best to engage with patients and/or their parents to facilitate patient involvement with the different stages of the COS development.

### Future Work

The consensus meeting and follow up meeting with parents has resulted in a COS which recommends what to measure. However, if future research measures these outcomes in different ways it will still be difficult to compare studies. The next steps will involve consideration of how each of these outcomes should be defined and measured. For each outcome, definitions and measurement instruments will need to be reviewed, whether a validated tool already exists and what methods have been used to measure this outcome in previous studies, as described in the systematic review (S2 Table).

For each of the outcomes included in the recommended COS this will include: consideration of methods of assessing hearing that might be influenced by the intervention, for example, differing methods depending on ventilation tube or hearing aid use; agreeing a definition of COM and methods of measurement; determining which aspects of speech development should be measured and identifying whether methods of measurement are already available; reviewing methods for assessment of receptive language, psychosocial development, AOM, listening skills, cholesteatoma and otalgia; establishing the most appropriate way to measure side effects of treatment; consideration of the impact of patient age group on the chosen method of assessment. With the exception of “listening skills” all outcomes have been measured in one or more of the studies identified in the systematic review.

Guidelines for the selection of outcome measurement instruments to be included in a COS are being developed by the Core Outcome Measurement Instrument Selection (COMIS) project [76] and will be consulted when available. Furthermore, the UK Cleft Audit means that for some outcomes there are potentially methods of measurement that have already been agreed by health professionals providing cleft care in the UK [77,78].

Our study recommends a core outcome set but also acknowledges that further work is needed to identify agreed methods of measurement for each of the outcomes as this is beyond the scope of the current study. As part of the methods development process consideration will be given to the age at which outcome assessments are appropriate and this will inform the length of follow up needed in a given trial.

The length of follow up will not be mandated and a similar approach will be adopted as by the OMERACT group where, if appropriate, outcomes will only be considered core should they be appropriate to the age group of participants and duration of follow up. For example, the OMERACT core outcome set includes one outcome which is only relevant should the duration of follow up be greater than 52 weeks[79]. The MOMENT study has involved multiple key stakeholder groups from the UK to ensure that a COS is suitable and well accepted in future research. However, to promote good uptake of the COS into future studies international consensus is needed. Cleft organisations exist in both Europe (The European Cleft Organisation) and the United States (American Cleft Palate-Craniofacial Association) and we plan to work with COMET [80]to pursue engagement of international health professionals through their membership.

It should also be noted that a COS is not static and should be revised or updated as new information becomes available. Should future trials using the COS recommended in this paper identify difficulties in its application then review of the included outcomes would be warranted.

Whilst OME affects around 75% of children with CP, it is also a common condition for children without cleft, with almost a fifth of children aged 1–5 years affected [1]. The COS described in the current study includes outcomes that have been identified from previous studies in both cleft and non-cleft populations suggesting that they may also be of relevance to studies of OME in children without CP. Further work in this area and engagement with stakeholders is warranted.

## Supporting Information

S1 Fig Scoring categories for health care professionals, parents, children and young people. (TIF)

S2 Fig Retrieved studies flow chart. (TIF)

S3 Fig Average scores in round 1 across all outcomes by stakeholder group. Shaded bars represent those who provided scores in round 1 only, open bars represent those scoring in both rounds 1 and 2.
(TIF)

S4 Fig Average scores in round 2 across all outcomes by stakeholder group. Shaded bars represent those who provided scores in round 2 only, open bars represent those scoring in both rounds 2 and 3.
(TIF)

S1 Table Definition of consensus. (DOCX)

S2 Table Characteristics of included papers including outcomes used. (DOCX)

S3 Table Breakdown of participants invited and completing all three rounds of the Delphi. (DOCX)

S4 Table Breakdown of response rate in each round by health professional group. (DOCX)

S5 Table Summary of outcomes discussed at consensus meeting. (DOCX)

S6 Table Stakeholder Representation at consensus meeting. (DOCX)
