# Supplementary figures and images for: The Importance of Integration of Stakeholder Views in Core Outcome Set Development: Otitis Media with Effusion in Children with Cleft Palate
Source: PLoS One. 2015 Jun 26;10(6):e0129514. doi: 10.1371/journal.pone.0129514 (PMC4483230; doi:10.1371/journal.pone.0129514)

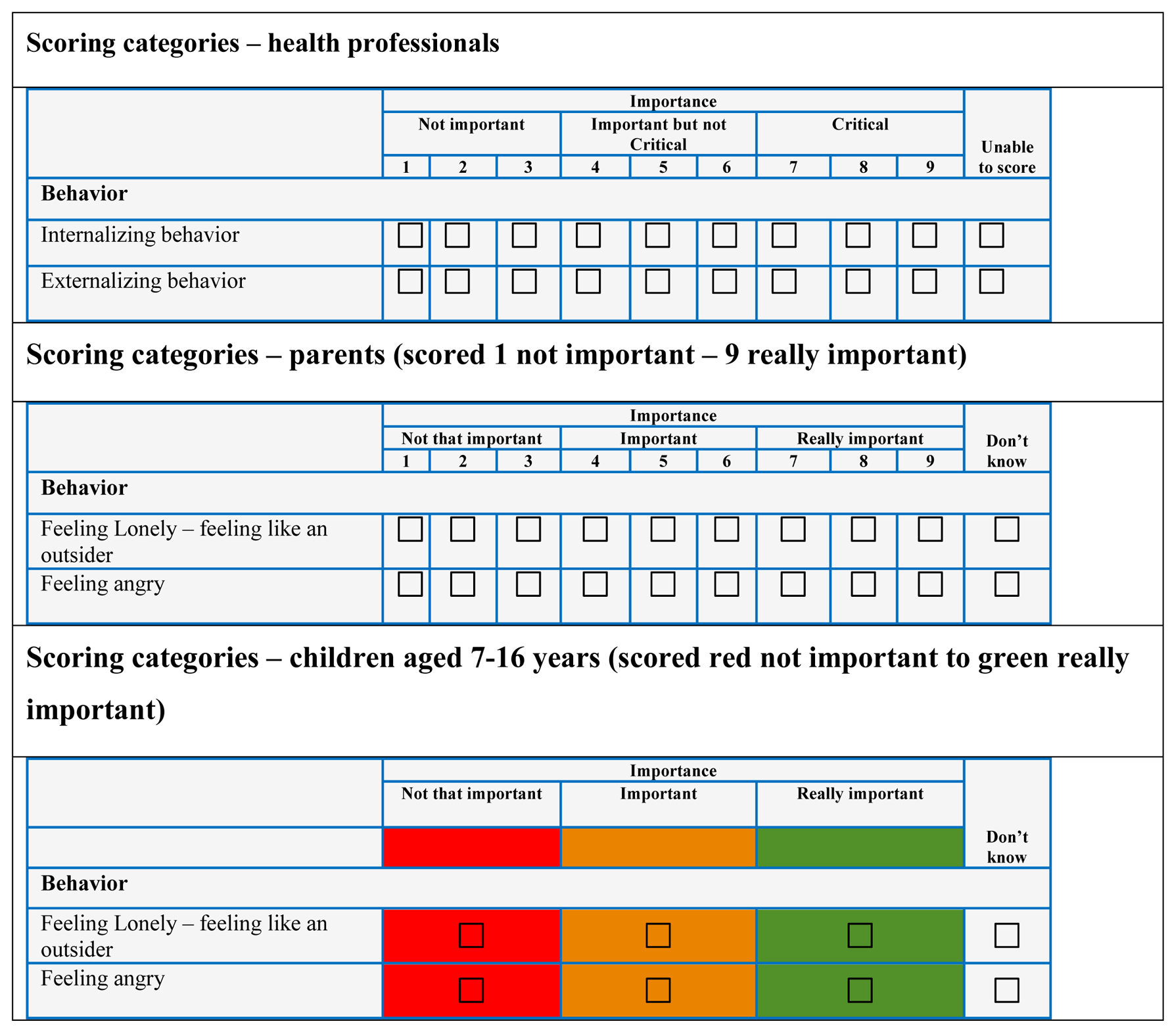

Supplement: S1 Fig — (TIF) [file pone.0129514.s001.tif]

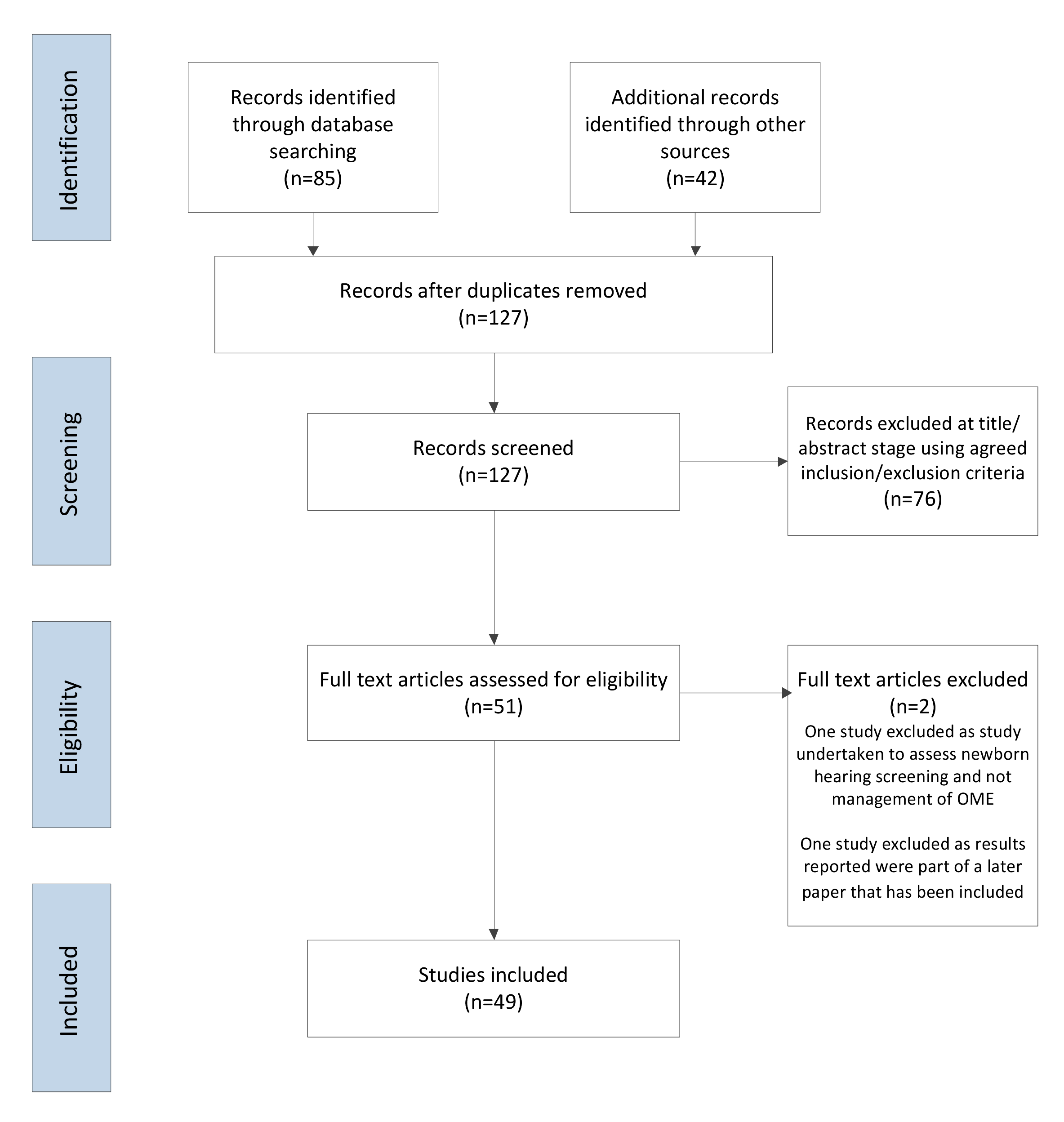

Supplement: S2 Fig — (TIF) [file pone.0129514.s002.tif]

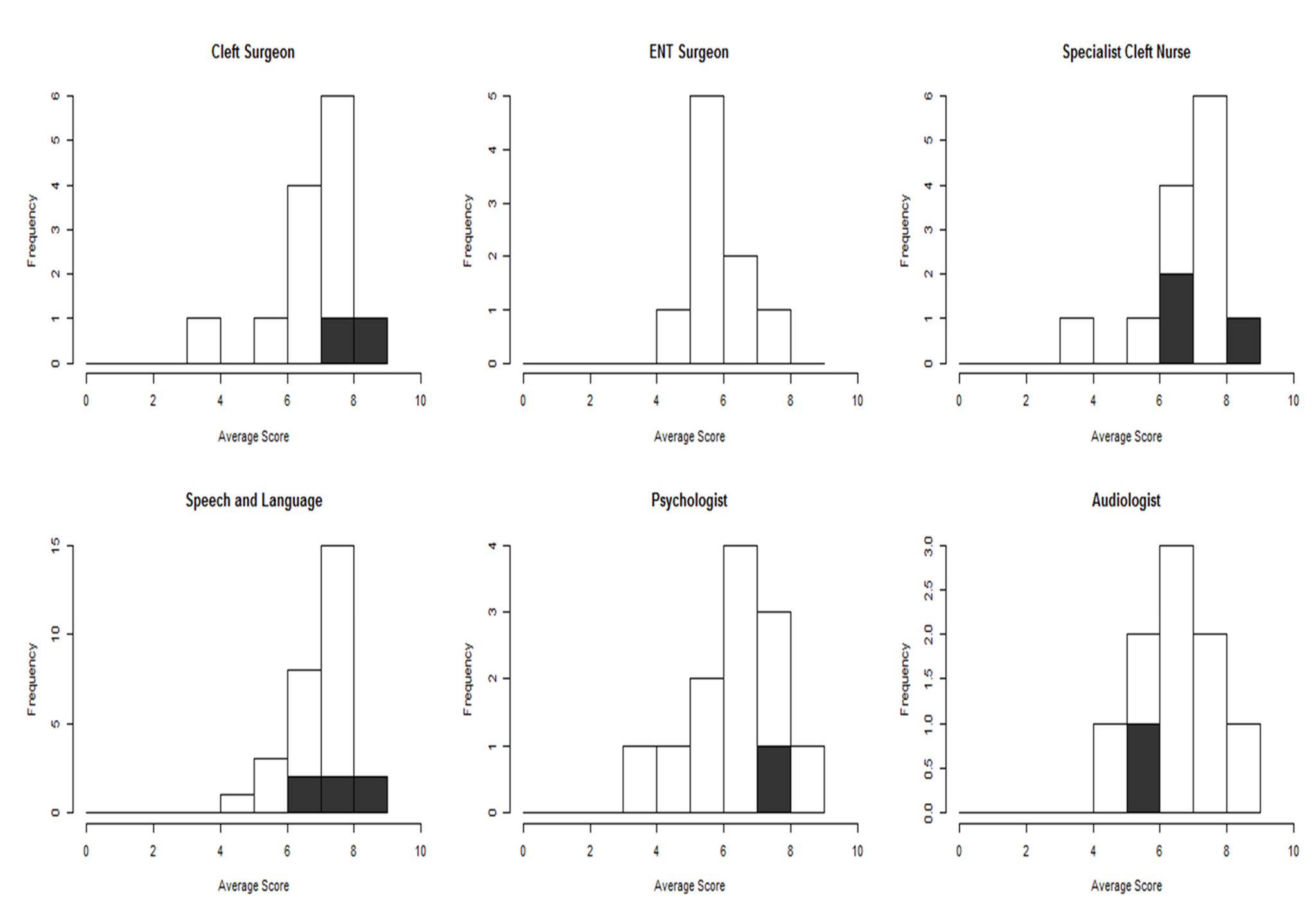

Supplement: S3 Fig — Shaded bars represent those who provided scores in round 1 only, open bars represent those scoring in both rounds 1 and 2. (TIF) [file pone.0129514.s003.tif]

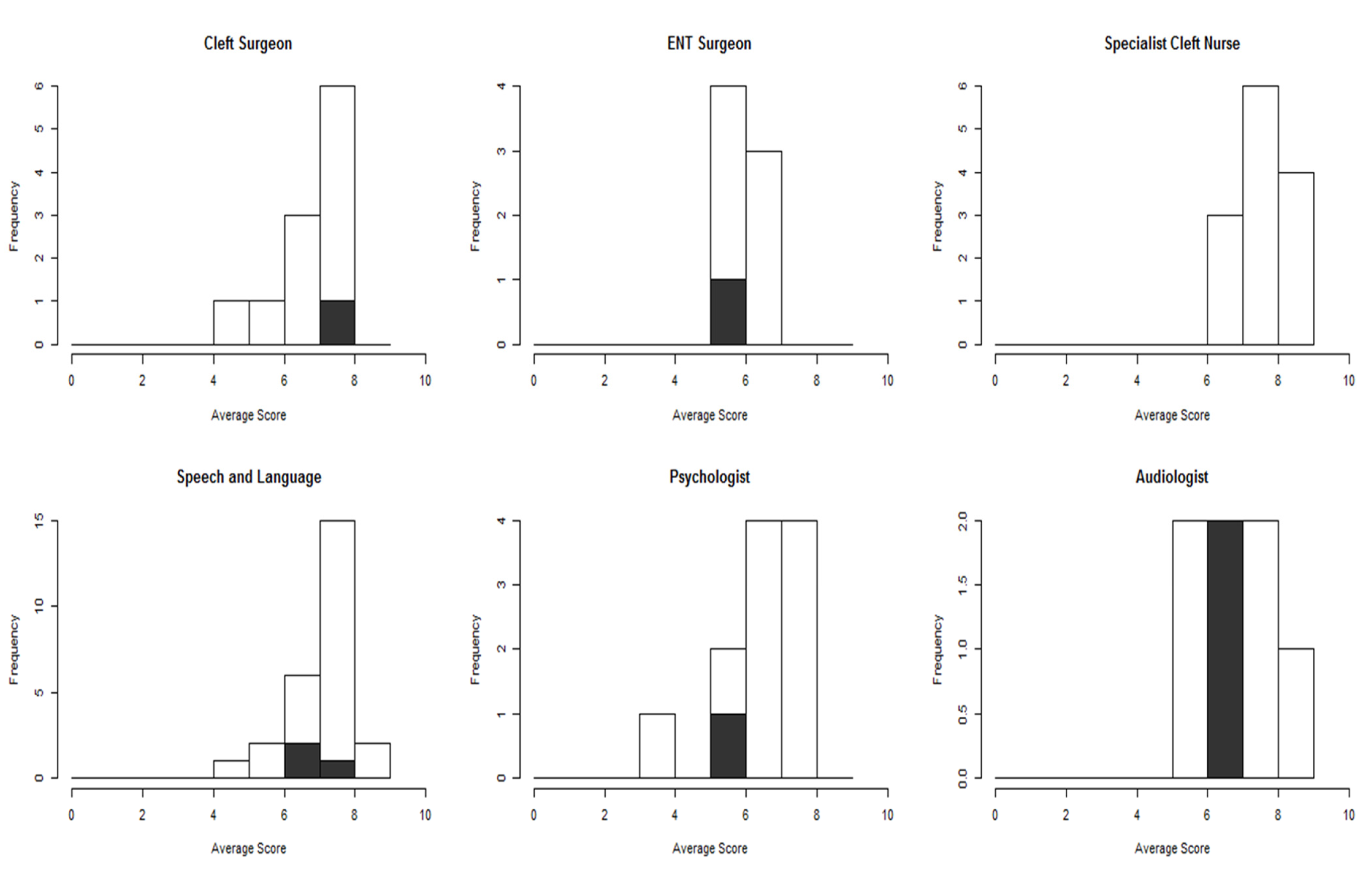

Supplement: S4 Fig — Shaded bars represent those who provided scores in round 2 only, open bars represent those scoring in both rounds 2 and 3. (TIF) [file pone.0129514.s004.tif]
