# Supplementary material for: The Importance of Integration of Stakeholder Views in Core Outcome Set Development: Otitis Media with Effusion in Children with Cleft Palate
Source: PLoS One. 2015 Jun 26;10(6):e0129514. doi: 10.1371/journal.pone.0129514 (PMC4483230; doi:10.1371/journal.pone.0129514)
Supplement: S1 Table — (DOCX) [file pone.0129514.s005.docx]

| **S1 Table. Definition of consensus** | | |
| --- | --- | --- |
| **Consensus classification** | **Description** | **Definition** |
| Consensus in | Consensus that outcome should be included in the core outcome set | 70% or more participants scoring as 7 to 9 AND <15% participants scoring as 1 to 3 |
| Consensus out | Consensus that outcome should not be included in the core outcomes set | 70% or more participants scoring as 1 to 3 AND <15% of participants scoring as 7 to 9 |
| No consensus | Uncertainty about importance of outcome | Anything else |
