# Supplementary material for: The Importance of Integration of Stakeholder Views in Core Outcome Set Development: Otitis Media with Effusion in Children with Cleft Palate
Source: PLoS One. 2015 Jun 26;10(6):e0129514. doi: 10.1371/journal.pone.0129514 (PMC4483230; doi:10.1371/journal.pone.0129514)
Supplement: S2 Table — (DOCX) [file pone.0129514.s006.docx]

| **S2 Table. – characteristics of included papers including outcomes used.** | | | | | | | | | |
| --- | --- | --- | --- | --- | --- | --- | --- | --- | --- |
| Study Number | Author (year) | Study Type | Duration of Follow up | Sample size | Participants | Interventions | Outcomes measured (*primary outcome) | Method of measurement | Comments |
| 1. | Moller (1982) | Retrospective Cohort | 12 months- 4 years. | 70 | Children with CP aged 12-156 months at study entry | CP with bilateral VT, Unilateral VT, anterior quadrant VT | Incidence of otorrhoea | Incidence of otorrhoea within 1 year of VT |  |
|  |  |  |  |  |  |  | Necessity to remove VT | Necessity to remove VT |  |
|  |  |  |  |  |  |  | Middle ear ventilation | Position of tympanic membrane (measure of middle ear ventilation) |  |
|  |  |  |  |  |  |  | Tympanosclerosis | Tympanosclerosis |  |
|  |  |  |  |  |  |  | Middle ear ventilation | Retraction |  |
|  |  |  |  |  |  |  | Necessity for new VT | Necessity for new VT |  |
|  |  |  |  |  |  |  | OME* | Otoscopic findings (OME) |  |
| 2. | Potsic (1979) | Retrospective Cohort | 5 years | 69 | Children with cleft palate. | No treatment vs VT when needed vs VT early insertion. | Hearing impairment* | Pure tone audiometry | Hearing impairment included as an outcome with two methods of assessment. |
|  |  |  |  |  |  |  | Hearing impairment* | Speech audiometry |  |
|  |  |  |  |  |  |  | OME | Otoscopic findings |  |
| 3. | Paradise and Bluestone (1974) | Case Series | 32 Months | 138 | Children with cleft palate aged <24months. Pittsburgh USA. | n/a | Incidence of otorrhoea | Otorrhoea – 6 months post op | OME included as an outcome with two methods of assessment. |
|  |  |  |  |  |  |  | Duration of otorrhoea |  |  |
|  |  |  |  |  |  |  | OME* | Pneumatic otoscopy |  |
|  |  |  |  |  |  |  | OME* | Microscopy |  |
|  |  |  |  |  |  |  | Perforation | Perforation |  |
|  |  |  |  |  |  |  | tympanic membrane  on otoscopy | Fullness or bulging of the tympanic membrane |  |
|  |  |  |  |  |  |  | tympanic membrane on  otoscopy | Erythema or colour of t  the tympanic membrane |  |
| 4. | Moller (1981) | Prospective Cohort | 36 Months | 261 | Children with cleft palate aged 1 month – 20 years (mean 7 years). Western and Northern Norway. | 28 bilateral VT, 40 unilateral VT | Hearing impairment* | Pure tone audiometry |  |
|  |  |  |  |  |  |  | Middle ear pressure | tympanometry |  |
|  |  |  |  |  |  |  | OME | Otoscopic findings |  |
|  |  |  |  |  |  |  | Scarring of the tympanic membrane | Otoscopic findings |  |
|  |  |  |  |  |  |  | Atelectasis of the tympanic membrane | Otoscopic findings |  |
|  |  |  |  |  |  |  | Tympanosclerosis of the tympanic membrane | Otoscopic findings |  |
|  |  |  |  |  |  |  | Acute Otitis media | Otoscopic findings |  |
|  |  |  |  |  |  |  | Chronic perforation | Otoscopic findings |  |
|  |  |  |  |  |  |  | Stapedial reflex | tympanometry |  |
| 5. | Smith *et al*. (1994) | Case Series, | 65 months | 81 | Children with cleft palate, age not stated. North Carolina, USA. | - | Eustachian tube dysfunction* | Tympanometry |  |
|  |  |  |  |  |  |  | Number of VTs until normal tympanometry | Tympanometry/note review |  |
|  |  |  |  |  |  |  | Average time to extrusion of VT | Note review |  |
|  |  |  |  |  |  |  | Hearing | Pure Tone Audiometry |  |
|  |  |  |  |  |  |  | Perforation of the tympanic membrane | Otoscopy |  |
| 6. | Hormann *et al*. (1991) | Prospective Cohort | Age 8 and 16 years | 184 | Children with cleft palate. Hamburg Germany (n=126) and Ioqa, USA (n=58) | Grommets vs early grommets | Risk of otorrhoea | Risk of otorrhoea |  |
|  |  |  |  |  |  |  | Middle ear status | Unknown |  |
| 7. | Broen *et al*. (1995) | Prospective cohort | 3 monthly follow up for 9-30 months | 28 (CP)  29) non-CP) | children with and without CP. Minnesota, USA. | Cleft vs non cleft | Hearing impairment* | Sound field audiometry | Hearing impairment included as an outcome with three methods of assessment. |
|  |  |  |  |  |  |  | Hearing impairment* | Visual reinforcement audiometry |  |
|  |  |  |  |  |  |  | Hearing impairment* | Pure Tone Audiometry |  |
|  |  |  |  |  |  |  | Middle ear function | Tympanometry |  |
|  |  |  |  |  |  |  | Hearing screening failures |  |  |
|  |  |  |  |  |  |  | Number of VTs | Audiometry number of ventilation tubes |  |
| 8. | Frable *et al*. (1985) | Case series | 6 monthly follow up for 10 years. | 36 | Children with cleft palate | CP children with bilateral VTs placed | Number of ventilation tubes | Note review |  |
|  |  |  |  |  |  |  | tympanic membrane atresia* | Otoscopic findings |  |
|  |  |  |  |  |  |  | Chronic otitis media* | Otoscopic findings |  |
|  |  |  |  |  |  |  | Incidence of acute otitis media | Parent report |  |
|  |  |  |  |  |  |  | Otitis media with Effusion | Otoscopic findings |  |
|  |  |  |  |  |  |  | Hearing Loss | Audiometry |  |
| 9. | Sheahan *et al*. (2002) ([32](#_ENREF_32)) | Case series | Mean follow up 6 years and 11 months. | 104 | children with cleft palate. Dublin, Ireland. | CP children treated for OME | chronic otitis media* | Otoscopic findings | Cholesteatoma, retraction and perforation have been grouped in this paper and are considered to be signs of chronic otitis media |
|  |  |  |  |  |  |  | Cholesteatoma | Otoscopic findings |  |
|  |  |  |  |  |  |  | Retraction | Otoscopic findings |  |
|  |  |  |  |  |  |  | Perforation | Otoscopic findings |  |
|  |  |  |  |  |  |  | Otitis Media with Effusion | Otoscopic findings |  |
|  |  |  |  |  |  |  | Hearing impairment | Pure tone audiometry |  |
| 10. | Hubbard *et al*. (1985) | Retrospective study | 60-132 months. | 48 | Children with cleft palate aged 3 months at study entry. Pittsburgh, USA. | VT at 3 months v VT 30 months. | Scarring of the tympanic membrane* | Otoscopy |  |
|  |  |  |  |  |  |  | Hearing loss | Pure Tone Audiometry |  |
|  |  |  |  |  |  |  | Hearing loss | Speech reception thresholds |  |
|  |  |  |  |  |  |  | Middle ear pressure | Pneumatic otoscopy |  |
|  |  |  |  |  |  |  | Middle ear pressure | tympanometry |  |
|  |  |  |  |  |  |  | Otitis media with effusion | Otoscopic findings |  |
|  |  |  |  |  |  |  | Hypernasality of speech | 5 point scale completed by speech and language therapist |  |
|  |  |  |  |  |  |  | Consonant articulation | Test of articulation |  |
|  |  |  |  |  |  |  | Social maturity | Vineland Social maturity scale |  |
|  |  |  |  |  |  |  | Social maturity | Wechsler intelligence scale |  |
|  |  |  |  |  |  |  | Self esteem | Cooper smith self-esteem inventory |  |
|  |  |  |  |  |  |  | Behaviour | Child behaviour checklist |  |
|  |  |  |  |  |  |  | Nasal resonance | Assessment by speech and language therapist |  |
|  |  |  |  |  |  |  | Perforation | Otoscopic findings |  |
| 11. | Gordon *et al.* (1988) | Retrospective study | Follow up 10-16 years post treatment. | 50 | Children with cleft palate. New Zealand | Ventilation tubes vs no treatment | Hearing impairment | Pure tone audiometry |  |
|  |  |  |  |  |  |  | Middle ear pressure | tympanometry |  |
|  |  |  |  |  |  |  | Tympanosclerosis * | Otoscopy |  |
|  |  |  |  |  |  |  | Scarring of the tympanic membrane * | Otoscopy |  |
|  |  |  |  |  |  |  | Retraction | Otoscopy |  |
|  |  |  |  |  |  |  | OME * | Otoscopy |  |
|  |  |  |  |  |  |  | Otorrhoea * | Otoscopy |  |
|  |  |  |  |  |  |  | Cholesteatoma * | Otoscopy |  |
| 12. | Robson *et al.* (1992) | Retrospective | 30-60month follow up | 74 | Children with cleft palate. Bristol, UK. | Ventilation tubes vs no treatment. | Otorrhoea | Otoscopy | Communication disorder (verbal comprehension and expression as an outcome with four methods of assessment. |
|  |  |  |  |  |  |  | Perforations of the tympanic membrane | Otoscopy |  |
|  |  |  |  |  |  |  | Tympanosclerosis | Otoscopy |  |
|  |  |  |  |  |  |  | Episodes of otalgia |  |  |
|  |  |  |  |  |  |  | Middle ear pressure | tympanometry |  |
|  |  |  |  |  |  |  | Atelectasis of the tympanic membrane | Otoscopy |  |
|  |  |  |  |  |  |  | Retraction of the tympanic membrane | Otoscopy |  |
|  |  |  |  |  |  |  | Velopharyngeal insufficienty | SLT assessment |  |
|  |  |  |  |  |  |  | OME | Otoscopy |  |
|  |  |  |  |  |  |  | Hearing impairment | Pure tone audiogram |  |
|  |  |  |  |  |  |  | Educational performance | Parent questionnaire (is child above/below average) |  |
|  |  |  |  |  |  |  | Level of speech therapy support required | Level of speech therapy support required |  |
|  |  |  |  |  |  |  | Behaviour | Parent questionnaire (is child above/below average) |  |
|  |  |  |  |  |  |  | Communication disorder (verbal comprehension and expression)* | Cleft related articulation at SLT assessment |  |
|  |  |  |  |  |  |  | Communication disorder (verbal comprehension and expression)* | Phonological problem at SLT assessment |  |
|  |  |  |  |  |  |  | Communication disorder (verbal comprehension and expression)* | language difficulties at SLT assessment |  |
|  |  |  |  |  |  |  | Communication disorder (verbal comprehension and expression)* | Learning difficulties at SLT assessment |  |
| 13. | Greig *et al. (1*999) | Case Series | 60 months. | 36 | Children with cleft palate. London, England. | CP children with bilateral VT tubes – | Hearing improvement | Parental questionnaire |  |
|  |  |  |  |  |  |  | Change in otorrhea | Parental questionnaire |  |
|  |  |  |  |  |  |  | Parental satisfaction with VT treatment * | Parental questionnaire |  |
|  |  |  |  |  |  |  | Speech improvement | Parental questionnaire |  |
|  |  |  |  |  |  |  | Receptive language | SLT assessment |  |
|  |  |  |  |  |  |  | Expressive language | SLT assessment |  |
|  |  |  |  |  |  |  | Speech development | SLT assessment |  |
|  |  |  |  |  |  |  | Global development | Unclear |  |
|  |  |  |  |  |  |  | Hearing impairment | Audiometry (not details given) |  |
|  |  |  |  |  |  |  | Nasal escape | SLT assessment |  |
|  |  |  |  |  |  |  | Cleft Speech | SLT assessment |  |
|  |  |  |  |  |  |  | Perforation | Presume otoscopy |  |
| 14. | Shaw *et al.* (2003) ([37](#_ENREF_37)) | Retrospective study | 10 years | 72 | Children with cleft palate. Liverpool, UK. | Children grouped into LAHSAL cleft classification | Speech – resonance * | SLT assessment |  |
|  |  |  |  |  |  |  | Speech –articulation * | SLT assessment |  |
|  |  |  |  |  |  |  | Number of grommets | Number of grommets |  |
| 15. | Freeland *et al.* (1981) | Retrospective study | Followed 6 monthly for 48 months. | 68 | Children with cleft palate. Oxford, UK | Recruited at birth with regular follow up. | OME | Otoscopic findings | Cholesteatoma, retraction and perforation have been grouped in this paper and are considered to be signs of chronic otitis media (Sheahan 2002) |
|  |  |  |  |  |  |  | Retraction* | Otoscopic findings |  |
|  |  |  |  |  |  |  | Perforation * | Otoscopic findings |  |
|  |  |  |  |  |  |  | Tympanosclerosis of the tympanic membrane | Otoscopic findings |  |
|  |  |  |  |  |  |  | language development | Reynell Developmental Lanaguage Score (RDLS) |  |
| 16. | Zheng *et al.* (2003) | Randomised Control Trial | 6 month post op (VTs), 20 month post op (control) | 62 | Children with cleft palate. China. | Palatoplasty (n=24) v palatoplasty +VT (n=38) | Presence of OME* | Unknown | Article in Chinese. Abstract and Ponduri 2009 review used. |
|  |  |  |  |  |  |  | Hearing levels | Unknown |  |
| 17. | Liu *et al.* (2004) | Case Series | 2weeks – 18 months post operatively. | 19 | Children with cleft palate. China | unilateral VT vs other ear as control with no VT | Complications | Unknown | Article in Chinese. Abstract and Ponduri 2009 review used. |
|  |  |  |  |  |  |  | Hearing loss* | Audiometry |  |
|  |  |  |  |  |  |  | Middle ear status | Unknown |  |
| 18. | Tanpowpong and Kittimanont (2007) | Cohort | 10 months | 23 | 6 children with CP and 17 without CP. Bangcok. | Myringotomy and VT insertion | Hearing impairment* | Pure Tone Audiometry |  |
|  |  |  |  |  |  |  | Middle Ear Pressure | Tympanometry |  |
|  |  |  |  |  |  |  | Otorheaa | Otoscopy |  |
|  |  |  |  |  |  |  | Time to extrusion of VTs | Note review |  |
| 19. | Civelek *et al.* (2007) | Retrospective | 72 month follow up. | 41 | 56 children with cleft palate, 15 children without cleft palate and history of VT insertion. Turkey. | CP vs non-CP with VTs | Perforation* | Otoscopic findings |  |
|  |  |  |  |  |  |  | Tympanosclerosis * | Otoscopic findings |  |
|  |  |  |  |  |  |  | Cholesteatoma* | Otoscopic findings |  |
|  |  |  |  |  |  |  | Retraction* | Otoscopic findings |  |
|  |  |  |  |  |  |  | Hearing impairment* | Pure Tone Audiometry |  |
|  |  |  |  |  |  |  | Middle ear pressure | Tympanometry |  |
|  |  |  |  |  |  |  | Velopharyngeal insufficieny | Speech assessment (method not specified) |  |
| 20. | Phua *et al.* (2009) ([24](#_ENREF_24)) | Retrospective | Minimum 2 years, maximum 15 years. | 234 | Children with cleft palate. Auckland, New Zealand. | Vt (45) v no treatment(189) | Hearing Loss* | Pure Tone Audiogram |  |
|  |  |  |  |  |  |  | Recurrent AOM | Note review |  |
|  |  |  |  |  |  |  | Persistent OME | Otoscopic findings |  |
|  |  |  |  |  |  |  | Retraction | Otoscopic findings |  |
|  |  |  |  |  |  |  | Perforation | Otoscopic findings |  |
|  |  |  |  |  |  |  | Cholesteatoma | Otoscopic findings |  |
|  |  |  |  |  |  |  | Subjective Hearing Loss | Note review |  |
|  |  |  |  |  |  |  | Number of VTs | Note review |  |
| 21 | Reiter *et al.* (2009) | Retrospective | 6 years | 116 | Children with cleft palate. Germany. | Divided age and type of cleft then +/- VTs. | Cholesteatoma * | Otoscopic findings |  |
|  |  |  |  |  |  |  | Hearing Loss | Pure tone audiogram |  |
|  |  |  |  |  |  |  | Middle ear pressure | Tympanometry |  |
|  |  |  |  |  |  |  | Atelectasis of the tympanic membrane* | Otoscopic findings |  |
|  |  |  |  |  |  |  | Perforations of the tympanic membrane * | Otoscopic findings |  |
|  |  |  |  |  |  |  | Retraction of the tympanic membrane * | Otoscopic findings |  |
|  |  |  |  |  |  |  | OME | Otoscopic findings |  |
| 22 | Szabo *et al.* (2010) | Retrospective | 5 years | 86 | Children with cleft palate. Connecticut, USA. | All cases VT | Hearing* | Newborn hearing screening |  |
|  |  |  |  |  |  |  | Number of surgeries | Note review |  |
|  |  |  |  |  |  |  | Atelectasis of the tympanic membrane | Otoscopic findings |  |
|  |  |  |  |  |  |  | Perforations of the tympanic membrane* | Otoscopic findings |  |
|  |  |  |  |  |  |  | Retraction of the tympanic membrane | Otoscopic findings |  |
|  |  |  |  |  |  |  | Tympanosclerosis | Otoscopic findings |  |
|  |  |  |  |  |  |  | Scarring of the tympanic membrane | Otoscopic findings |  |
| 23 | Hornigold *et al.* (2008) | Long term follow up data of RCT | 20 years post VT insertion in original RCT. | 7 | Children with cleft palate who had participated in previous RCT. United Kingdom | VT insertion v control | Hearing loss | Pure Tone Audiometry | In this study primary outcomes (* and bold) were grouped in the paper as symptomatology |
|  |  |  |  |  |  |  | Middle ear function | Tympanometry |  |
|  |  |  |  |  |  |  | Mucosal COM | Otoscopic findings |  |
|  |  |  |  |  |  |  | Cholesteatoma (squamous COM) | Otoscopic findings |  |
|  |  |  |  |  |  |  | Otorrhea * | Patient interview |  |
|  |  |  |  |  |  |  | Subjective hearing loss* | Patient interview |  |
|  |  |  |  |  |  |  | Otalgia* | Patient interview |  |
|  |  |  |  |  |  |  | Vertigo* | Patient interview |  |
|  |  |  |  |  |  |  | Tinnitus* | Patient interview |  |
|  |  |  |  |  |  |  | Need for further surgery* | Patient interview |  |
| 24 | Merrick *et al.*  (2007) | Cohort study | Mean 8 years of age. Follow up post palatoplasty. | 100 | 50 children with cleft palate, 50 children without cleft palate. United Kingdom. | VT (50) v control (50) | OME | Otoscopy |  |
|  |  |  |  |  |  |  | Speech intelligibility* | Cleft palate speech assessment audit form |  |
|  |  |  |  |  |  |  | Speech nasality* | assessment audit form |  |
|  |  |  |  |  |  |  | Nasal air flow* | assessment audit form |  |
|  |  |  |  |  |  |  | Consonant production * | assessment audit form |  |
|  |  |  |  |  |  |  | Cleft type characteristics* | assessment audit form |  |
| 25 | Curtin *et al.* (2009) | Prospective cohort | 6 months post palate repair | 33 | Children with cleft palate. Stanford, USA. | VT at 9 months with a 6 month follow up. | Incidence of otorrhea* | Parent report | Hearing impairment as an outcome with three methods of assessment. |
|  |  |  |  |  |  |  | VT patency | Tympanometry |  |
|  |  |  |  |  |  |  | Hearing impairment | Behavioural audiometry |  |
|  |  |  |  |  |  |  | Hearing impairment | Sound field audiometry |  |
|  |  |  |  |  |  |  | Hearing impairment | New-born infant hearing screen |  |
| 26 | Mandel *et al.* (1992) | RCT | 4 weeks. | 111 | Children without cleft palate. Pittsburgh, USA. | Bi VT vs Bi Myringotomy vs no surgery | Middle ear effusion * | Pneumatic otoscopy |  |
|  |  |  |  |  |  |  | Middle ear pressure | Tympanometry |  |
|  |  |  |  |  |  |  | Recurrence of OME following resolution | Note review |  |
|  |  |  |  |  |  |  | Incidence of acute otitis media | Note review |  |
|  |  |  |  |  |  |  | Hearing impairment | Age appropriate hearing test (procedures varied according to age) |  |
|  |  |  |  |  |  |  | Adverse events | Incidence of hyperactivity, increased appetite, vomiting, irritability, diarrhoea , abdominal discomfort, rash. |  |
| 27 | Casselbrant *et al.* (2009) | RCT | Up to 36 months | 98 | Children without cleft palate. Pittsburgh, USA. | myringotomy +VT (32), adenoids with myringotomy + VT (32) adenoidectomy with myringotomy (34) | Percentage of time with OME * | Pneumatic otoscopy | Percentage of time with OME as an outcome with three methods of assessment. |
|  |  |  |  |  |  |  | Percentage of time with OME * | Tympanometry |  |
|  |  |  |  |  |  |  | Percentage of time with OME | Otoscopy |  |
|  |  |  |  |  |  |  | Requirement for further surgery | Note review |  |
|  |  |  |  |  |  |  | Incidence of acute otitis media | Outpatient assessment |  |
|  |  |  |  |  |  |  | Incidence of otorrhea | Outpatient assessment |  |
|  |  |  |  |  |  |  | VT functional status | Tympanometry |  |
|  |  |  |  |  |  |  | Perforation of the tympanic membrane | Otoscopy |  |
| 28 | Koivunen *et al. (*2004) | RCT | 2 years | 180 | Children without cleft palate. Oulu, Finland | adenoidectomy (60) chemoprohpylaxis (60) placebo (60) | Intervention failure* | 2 acute episodes in 2 months or 3 in 6 months based on symptom diary  Or  Middle ear effusion for at least 2 months as assessed by study otolaryngologist using pneumatic otoscope | Necessity to visit doctor – definition of doctor not specified in paper, would assume that the outcome describes unplanned visits to the GP and not planned study visits to the consultant |
|  |  |  |  |  |  |  | Incidence of acute otitis media | Symptom diary |  |
|  |  |  |  |  |  |  | Necessity to visit doctor | Symptom diary |  |
|  |  |  |  |  |  |  | Requirement for antibiotics | Symptom diary |  |
|  |  |  |  |  |  |  | Days with rhinitis | Symptom diary |  |
|  |  |  |  |  |  |  | Days with earache | Symptom diary |  |
|  |  |  |  |  |  |  | Days with fever | Symptom diary |  |
|  |  |  |  |  |  |  | Incidence of adverse events | Symptom diary |  |
| 29 | Matilla *et al.*  (2003) | RCT | Follow up until age 2, mean follow up 7 months. | 137 | Children without cleft palate. Helsinki, Finland. | Bilateral VTs (63) vs Bi lateral VTs +adenoidectomy (74) | Rate of acute otitis media * | Otoscopy | Rate of AOM as an outcome with two methods of assessment. |
|  |  |  |  |  |  |  | Rate of acute otitis media * | Symptomology |  |
|  |  |  |  |  |  |  | Rate of otits media episodes caused by *S pnemoniae* | Culture |  |
|  |  |  |  |  |  |  | Rate of otits media episodes caused by *H influenzae* | Culture |  |
|  |  |  |  |  |  |  | Rate of otits media episodes caused by *M catarrhalis* | culture |  |
|  |  |  |  |  |  |  | Middle ear pressure | Tympanometry |  |
|  |  |  |  |  |  |  | Number of days with otorrhea | Follow up card |  |
| 30 | Paradise *et al.*  (1990) | RCT  Cohort | 3 years | 99 in RCT  114 in cohort | children without cleft palate. Pittsburgh, USA. | adenoidectomy (99) vs control (114) | Proportion of time with otitis media | Interpolation of visit data. Pneumatic otoscopy and bi weekly enquiries by nurse |  |
|  |  |  |  |  |  |  | Number of VT insertions | Case note review |  |
|  |  |  |  |  |  |  | Number of days when experienced otalgia | Biweekly enquiries by nurse |  |
|  |  |  |  |  |  |  | Number of days receiving antibiotics | Biweekly enquiries by nurse |  |
| 31 | Paradise *et al.*  (1999) | RCT | Up to 3 years | 461 (304 in 3 way trial and 157 in 2 way trial) | children without cleft palate. Pittsburgh, USA. | adenoidectomy (n=100) vs adenotonsillectomy (n=180) vs control (n=181) | Number of episodes of acute otitis media* | Not specified |  |
|  |  |  |  |  |  |  | Proportion of time with otitis media | Interpolation of visit data. Pneumatic otoscopy and bi weekly enquiries by nurse |  |
|  |  |  |  |  |  |  | Number of VT insertions | Case note review |  |
|  |  |  |  |  |  |  | Number of days when experienced otalgia | Biweekly enquiries by nurse |  |
|  |  |  |  |  |  |  | Number of myringotomies | Not specified |  |
|  |  |  |  |  |  |  | Number of days receiving antibiotics | Biweekly enquiries by nurse |  |
| 32 | Rynnel-Dagoo *et al.* (1978) | Prospective controlled study | 24 months | 76 | Children without cleft palate. Sweden. | adenoidectomy (37) control (39) | Change in frequency of common cold | unknown |  |
|  |  |  |  |  |  |  | Change in frequency of purulent otitis media | Unknown |  |
|  |  |  |  |  |  |  | Change in frequency of serious otitis media | Unknown |  |
|  |  |  |  |  |  |  | Change in frequency of nasal obstruction | Unknown |  |
| 33 | Gates *et al.*  (1989) | RCT | 24 months | 491 | Children without cleft palate. Texas, USA. | bilateral myringotomy (107) vs Bilateral VTs (129), vs adenoidectomy (130) vs adenoidectomy and bilateral VTs (125) | Time with hearing loss | Unknown |  |
|  |  |  |  |  |  |  | Time with OME | Unknown |  |
|  |  |  |  |  |  |  | Time to recurrence of OME | Unknown |  |
|  |  |  |  |  |  |  | Requirement for further VT insertion. | Unknown |  |
| 34 | Hammaren-Malmi *et al.*  , (2005) ([51](#_ENREF_51)) | RCT | 12 months | 217 | Children without cleft palate. Helsinki, Finland. | adenoidectomy +bilateral VTs (109) vs bilateral VTs only (108) | Number of acute otitis media episodes in 12 months* | Patient diary and review by GP (primary care doctor) | IB and NH agreed only one outcome in paper |
| 35 | Roydhouse, (1980) | RCT | 36 months | 169 | Children without cleft palate. Auckland, New Zealand. | Bilateral VT + adenoids (50), bilat VT no adenoids (50), control (69) | Presence of OME | Unknown |  |
|  |  |  |  |  |  |  | Requirement for repeated grommets | Unknown |  |
|  |  |  |  |  |  |  | Number of relapses | Unknown |  |
| 36 | Black *et al.*  (1990) | RCT | 2 years | 149 | Children without cleft palate. Oxford, United Kingdom. | Adenoidectomy, myringotomy and VTs (37) vs adenoidectomy and VT, (38) vs myringotomy and VTs (37) vs VTs (37). | Hearing impairment * | Pure tone audiometry |  |
|  |  |  |  |  |  |  | Developmental progress | Parental opinion of child’s progress |  |
|  |  |  |  |  |  |  | Presence of an abnormal tympanogram | Impedance tympanometry |  |
|  |  |  |  |  |  |  | Adverse side effects of treatment | Parental opinion (favourable, uncertain or unfavourable) |  |
|  |  |  |  |  |  |  | Requirement for further surgery | Note review |  |
|  |  |  |  |  |  |  | Parents opinion on treatment | 3 point scale. |  |
| 37 | Dempster *et al* (1993) ([45](#_ENREF_45)) | RCT | 12 months | 78 | children without cleft palate. Glasgow, United Kingdom. | Adenoidectomy + VTs (37), vs VTs only VT(35) | Presence of OME * | Otoscopy | Presence of OME likely to be primary outcome but paper states two primary outcomes, presence of OME and hearing |
|  |  |  |  |  |  |  | Presence of OME * | Tympanometry |  |
|  |  |  |  |  |  |  | Hearing* | Pure tone audiometry |  |
|  |  |  |  |  |  |  | Tympanosclerosis | Otoscopy |  |
|  |  |  |  |  |  |  | Perforation | Otoscopy |  |
|  |  |  |  |  |  |  | Retraction | Otoscopy |  |
| 38 | Maw *et al* (1999) | RCT | 18 months | 182 | Children without cleft palate. Bristol, United Kingdom. | adenoidectomy (47) vs adenoidectomy and tonsilectomy (47) vs control (56) | Hearing loss | Age appropriate hearing test | Expressive language, Verbal comprehension (grouped as language development) |
|  |  |  |  |  |  |  | Expressive language* | Reynell test at 9 and 18 months |  |
|  |  |  |  |  |  |  | Verbal comprehension* | Reynell test at 9 and 18 months |  |
|  |  |  |  |  |  |  | Mental development | Griffiths mental development scales |  |
|  |  |  |  |  |  |  | Middle ear pressure | Tympanometry |  |
|  |  |  |  |  |  |  | Presence of OME | Otoscopy |  |
|  |  |  |  |  |  |  | Requirement for further VTs | Note review |  |
| 39 | Rach *et al.* (1991) | RCT | 6 months | 43 | Children without cleft palate. Netherlands. | VTs v no surgery | Language development – Verbal expression * | Reynell test |  |
|  |  |  |  |  |  |  | Language development – Verbal comprehension * | Reynell test |  |
|  |  |  |  |  |  |  | Middle ear pressure | Tympanometry |  |
|  |  |  |  |  |  |  | Duration of VT tube in situ | Tympanometry |  |
|  |  |  |  |  |  |  | Presence of OME | Tympanometry |  |
| 40. | Rovers *et al.* (2000) | RCT | 12 months | 187 | Children without cleft palate. Netherlands. | VT (93) v No surgery (94) | Expressive language* | Schlichting test |  |
|  |  |  |  |  |  |  | Expressive language* | Lexi test |  |
|  |  |  |  |  |  |  | Comprehensive language* | Reynell yest |  |
|  |  |  |  |  |  |  | Hearing loss | Visual reinforcement audiometry |  |
|  |  |  |  |  |  |  | IQ | Bayleys |  |
|  |  |  |  |  |  |  | OME | Otoscopy |  |
|  |  |  |  |  |  |  | OME | Tympanometry |  |
| 41. | Johnston *et al.* (2004) | Screening followed by RCT | Follow up to age 3 years. | 429 | Children without cleft palate. Pittsburg, USA. | VT (216) vs Delayed VT (213) | Tympanoscerosis* | Otoscopy | Primary outcome- tympanic membrane abnormalities which includes – tympanosclerosis, fibrosis, atrophy, retraction pocket, perforation and cholesteatoma |
|  |  |  |  |  |  |  | Fibrosis* | Otoscopy |  |
|  |  |  |  |  |  |  | Atrophy* | Otoscopy |  |
|  |  |  |  |  |  |  | Retraction pocket* | Otoscopy |  |
|  |  |  |  |  |  |  | Perforation * | Otoscopy |  |
|  |  |  |  |  |  |  | Hearing impairment | Pure tone audiometry |  |
|  |  |  |  |  |  |  | Cholesteatoma* | Otoscopy |  |
| 42. | Paradise *et al.* (2001) | RCT | Follow up to age 3 years. | 429 | Children without cleft palate. Pittsburg, USA. | VT (216) vs Delayed VT (213 | Cognition* | General cognitive index of McCarthy scales of children’s abilities |  |
|  |  |  |  |  |  |  | Hearing loss* | Age appropriate hearing tests |  |
|  |  |  |  |  |  |  | Behaviour* | child behaviour checklist |  |
|  |  |  |  |  |  |  | Receptive language* | Peabody-revised picture vocabulary test |  |
|  |  |  |  |  |  |  | expressive language* | Number of different words |  |
|  |  |  |  |  |  |  | expressive language* | Percentage of consonants correct - revised |  |
|  |  |  |  |  |  |  | expressive language* | Mean length of utterance in morphemes |  |
|  |  |  |  |  |  |  | Parental distress* | Parental stress index, short form |  |
|  |  |  |  |  |  |  | Duration of OME | Pneumatic otoscopy |  |
|  |  |  |  |  |  |  | Duration of OME | tympanometry |  |
| 43. | Maw (1983) | RCT | 12 months | 222 | Children without cleft palate. Bristol, United Kingdom. | adenoidectomy (36) vs no surgery (33) vs adenotonsillectomy (34) | Presence OME* | Pneumatic otoscopy | Although other methodologies listed these were to confirm OME as part of patient screening and were not used as an outcome. |
| 44. | Zielhus *et al.* (1989) | Screening followed by RCT | Up to age 4 years | 43 | Children without cleft palate. Netherlands. | VT (22) vs no treatment (21) | Presence OME | tympanometry | Verbal comprehension and verbal expression grouped as language development |
|  |  |  |  |  |  |  | OME | Otoscopy |  |
|  |  |  |  |  |  |  | Verbal comprehension * | Reynell’s test |  |
|  |  |  |  |  |  |  | Verbal expression * | Reynell’s test |  |
| 45. | Fiellau-Nikolajsen *et al.* (1980) | RCT | 6 months postoperativelt | 42 | Children without cleft palate. Aarhus, Denmark. | myringtomy with adenoids (20) myringotomy only (22) | Middle ear pressure* | Tympanometry | Presence of middle ear reflexes was measured but not discussed in the paper |
|  |  |  |  |  |  |  | Presence of OME | otoscopy |  |
|  |  |  |  |  |  |  | Duration of OME | otoscopy (repeated over time) |  |
|  |  |  |  |  |  |  | Hearing impairment | Pure tone audiometry |  |
|  |  |  |  |  |  |  | Presence of middle ear reflexes | Impedance audiometry |  |
|  |  |  |  |  |  |  | Middle ear pressure* | Tympanometry |  |
| 46. | Nguyen *et al.* (2004) | RCT | Minimum 12 months | 63 | Children without cleft palate. Quebec, Canada | VTs and adenoidectomy (23) VTs only (40) | Recurrence Acute Otitis Media * | Patient questionnaire | Primary outcome included in paper as “treatment failure“ as defined by the three outcomes listed. |
|  |  |  |  |  |  |  | Recurrence of OME over 3 months * | Patient questionnaire |  |
|  |  |  |  |  |  |  | Re-insertion of Ventilation Tubes * | Patient questionnaire |  |
| 47. | Paradise *et al.* (2007) | RCT | Follow up at age 9 10 11 years. | 429 in original study. 391 in follow up. | children without cleft palate | VTs (216) v Delayed treatment (213) | Literacy | Woodcock reading mastery tests | Multilple methods of assessment for outcomes listed in paper.  Follow up of 2003 and 2005 paper |
|  |  |  |  |  |  |  | Literacy | Oral fluency -The number of words in a grade level passage read correctly |  |
|  |  |  |  |  |  |  | Literacy | Spelling and writing samples subtests of the Woodcock Johnson III tests of achievement |  |
|  |  |  |  |  |  |  | Phonological awareness | Ellison and Rapid Letter Naming subtests of the comprehensive tests of phonological processing |  |
|  |  |  |  |  |  |  | Auditory processing ability | Children’s version of the hearing in noise test |  |
|  |  |  |  |  |  |  | Attention, impulsivity and psychological functioning | Disruptive behaviours disorders rating scale |  |
|  |  |  |  |  |  |  | Attention, impulsivity and psychological functioning | children behaviour checklist |  |
|  |  |  |  |  |  |  | Attention, impulsivity and psychological functioning | impairment rating scales |  |
|  |  |  |  |  |  |  | Attention, impulsivity and psychological functioning | Social skills scale of the social skills rating system . |  |
|  |  |  |  |  |  |  | Intelligence and academic achievement | Wechsler abbreviated scale of intelligence |  |
|  |  |  |  |  |  |  | Intelligence and academic achievement | Calculation subtest of the Woodcock Johnson II test of achievement |  |
|  |  |  |  |  |  |  | Attention, impulsivity and psychological functioning | Continuous performance test – Visual |  |
|  |  |  |  |  |  |  | Attention, impulsivity and psychological functioning | Continuous performance test – auditory |  |
| 48. | Paradise *et al.* (2005) | RCT | Follow up at age 6 | 429 | Children without cleft palate. Pittsburg, USA | VT (216) v Delayed treatment (213) | Intelligence and academic achievement | Wechsler abbreviated scale of intelligence | Follow up of 2003 paper |
|  |  |  |  |  |  |  | Receptive vocabulary | Peabody picture vocabulary test |  |
|  |  |  |  |  |  |  | Behaviour | Parent reported inventories - Child behaviour checklist |  |
|  |  |  |  |  |  |  | Phonologic memory | Non word repetition task |  |
|  |  |  |  |  |  |  | Auditory processing and language | SCAN Test |  |
|  |  |  |  |  |  |  | Vocabulary diversity | Conversational sample – number of different words |  |
|  |  |  |  |  |  |  | Sentence length and grammatical complexity | Conversational sample – length of utterances in morphemes |  |
|  |  |  |  |  |  |  | Speech sound production | Conversational sample – Percentage of consonants correct |  |
|  |  |  |  |  |  |  | Parent-child stress | Parent reported inventories - Parental stress index |  |
| 49. | Paradise *et al.* (2003) | RCT | Follow up at age 4 years. | 429 | Children without cleft palate. Pittsburg, USA | VT (216) v Delayed treatment (213) | Cognition | McCarthys scales of children’s abilities |  |
|  |  |  |  |  |  |  | Receptive language | PPVT-R |  |
|  |  |  |  |  |  |  | Phonological memory | Non word repetition test |  |
|  |  |  |  |  |  |  | Expressive language | Word diversity (NDW) |  |
|  |  |  |  |  |  |  | Expressive language | Sentence length and grammatical complexity (MLUm) |  |
|  |  |  |  |  |  |  | Expressive language | Speech sound production (PCC-R) |  |
|  |  |  |  |  |  |  | Parental-child stress | Parental stress index |  |
|  |  |  |  |  |  |  | Behaviour | Child behaviour checklist |  |
