# Supplementary material for: The Importance of Integration of Stakeholder Views in Core Outcome Set Development: Otitis Media with Effusion in Children with Cleft Palate
Source: PLoS One. 2015 Jun 26;10(6):e0129514. doi: 10.1371/journal.pone.0129514 (PMC4483230; doi:10.1371/journal.pone.0129514)
Supplement: S3 Table — (DOCX) [file pone.0129514.s007.docx]

| **S3 Table: Breakdown of participants invited and completing all three rounds of the Delphi.** | | | | | | | | | | |
| --- | --- | --- | --- | --- | --- | --- | --- | --- | --- | --- |
| Site Number |  | Cleft Surgeon | ENT Surgeon | Geneticist | Specialist Cleft Nurse | Speech and Language Therapist | Psychologist | Audiologist/ Audiological physician | **TOTAL** | |
| 1 | Total invited for site | 4 | 3 | 1 | 2 | 3 | 2 | 3 | | **18** |
|  | Actual completing all three rounds | 0 | 1 | 0 | 0 | 0 | 0 | 0 | | **1** |
| 2 | Total invited for site | 3 | 1 | 0 | 1 | 5 | 1 | 2 | | **13** |
|  | Actual completing all three rounds | 1 | 0 | 0 | 0 | 1 | 0 | 1 | | **3** |
| 3 | Total invited for site | 1 | 0 | 0 | 3 | 10 | 0 | 1 | | **15** |
|  | Actual completing all three rounds | 1 | 1 | 0 | 3 | 6 | 0 | 0 | | **11** |
| 4 | Total invited for site | 1 | 2 | 1 | 4 | 6 | 3 | 0 | | **17** |
|  | Actual completing all three rounds | 0 | 0 | 0 | 0 | 1 | 1 | 0 | | **2** |
| 6 | Total invited for site | 2 | 0 | 2 | 3 | 5 | 3 | 3 | | **18** |
|  | Actual completing all three rounds | 1 | 0 | 0 | 1 | 1 | 2 | 2 | | **7** |
| 7 | Total invited for site | 2 | 0 | 2 | 3 | 2 | 4 | 4 | | **17** |
|  | Actual completing all three rounds | 1 | 0 | 0 | 0 | 1 | 3 | 0 | | **5** |
| 5 | Total invited for site | 1 | 1 | 2 | 0 | 1 | 1 | 1 | | **7** |
|  | Actual completing all three rounds | 0 | 0 | 0 | 0 | 0 | 1 | 0 | | **1** |
| 8 | Total invited for site | 2 | 0 | 1 | 2 | 2 | 0 | 1 | | **8** |
|  | Actual completing all three rounds | 0 | 1 | 0 | 0 | 0 | 0 | 1 | | **2** |
| 9 | Total invited for site | 2 | 2 | 1 | 3 | 2 | 0 | 1 | | **11** |
|  | Actual completing all three rounds | 0 | 2 | 0 | 0 | 2 | 0 | 1 | | **5** |
| 10 | Total invited for site | 5 | 0 | 0 | 6 | 4 | 2 | 0 | | **17** |
|  | Actual completing all three rounds | 0 | 0 | 0 | 3 | 2 | 1 | 0 | | **6** |
| 11 | Total invited for site | 2 | 1 | 1 | 3 | 6 | 2 | 2 | | **17** |
|  | Actual completing all three rounds | 0 | 0 | 0 | 1 | 2 | 0 | 0 | | **3** |
| 12 | Total invited for site | 2 | 0 | 1 | 2 | 3 | 2 | 2 | | **12** |
|  | Actual completing all three rounds | 2 | 1 | 0 | 1 | 3 | 1 | 1 | | **9** |
| 13 | Total invited for site | 4 | 2 | 1 | 5 | 10 | 3 | 2 | | **27** |
|  | Actual completing all three rounds | 1 | 0 | 0 | 2 | 3 | 2 | 0 | | **8** |
| 14 | Total invited for site | 2 | 1 | 0 | 5 | 3 | 0 | 1 | | **12** |
|  | Actual completing all three rounds | 1 | 1 | 0 | 2 | 1 | 0 | 0 | | **5** |
| 15 | Total invited for site | 3 | 0 | 1 | 2 | 0 | 1 | 1 | | **8** |
|  | Actual completing all three rounds | 2 | 0 | 0 | 0 | 1 | 0 | 1 | | **4** |
| 99 - registered as  "other site" | Actual completing all three rounds | 1 | 0 | 0 | 0 | 0 | 0 | 0 | | **1** |
|  | **TOTAL for all sites** | **37** | **13** | **14** | **44** | **62** | **24** | **24** | | **218** |
|  | **TOTAL completing round 3 for all sites** | **11** | **7** | **0** | **13** | **24** | **11** | **7** | | **73** |
