# Supplementary material for: The Importance of Integration of Stakeholder Views in Core Outcome Set Development: Otitis Media with Effusion in Children with Cleft Palate
Source: PLoS One. 2015 Jun 26;10(6):e0129514. doi: 10.1371/journal.pone.0129514 (PMC4483230; doi:10.1371/journal.pone.0129514)
Supplement: S4 Table — (DOCX) [file pone.0129514.s008.docx]

| **S4 Table: Breakdown of response rate in each round by health professional group.** | | | | | | | | | |
| --- | --- | --- | --- | --- | --- | --- | --- | --- | --- |
| Stakeholder Group | Cleft Surgeon | ENT Surgeon | Geneticist | Specialist Cleft Nurse | Speech and Language therapist | Paediatrician | Psychologist | Audiologist/ Audiological Physician | Total |
| **Round 1 response rates** | | | | | | | | | |
| Number invited to participate | 37 | 13 | 14 | 44^a^ | 62^b^ | 9 | 24^c^ | 15^d^ | 218 |
| Number of respondents | 15 | 9 | 3 | 18 | 36 | 3 | 13 | 7 | 104 |
| Percentage completing round 1 | 43% | 69% | 21% | 39% | 52% | 33% | 48% | 46% | 48% |
| **Round 2 response rates** | | | | | | | | | |
| Number invited to participate | 15 | 9 | 0^e^ | 17^f^ | 34^g^ | 0^h^ | 13 | 10 | 99 |
| Number of respondents | 13 | 9 | N/A | 14 | 28 | N/A | 12 | 9 | 85 |
| Percentage completing round 2 | 87% | 100% | N/A | 82% | 82% | N/A | 92% | 90% | 86% |
| **Round 3 response rates** | | | | | | | | | |
| Number invited to participate | 12^i^ | 8^j^ | 0^e^ | 13^k^ | 27^l^ | 0^h^ | 12 | 9 | 81 |
| Number of respondents | 11 | 7 | N/A | 13 | 24 | N/A | 11 | 7 | 73 |
| Percentage completing round 3 | 92% | 88% | N/A | 100% | 89% | N/A | 92% | 92 | 90% |
| 1. 47 invited but 3 with email failures after initial email invite 2. 69 invited but 7 with email failures or on maternity leave 3. 26 invited but 2 with email failures at time of initial email invite 4. 16 invited but 1 with email failure at time of initial email invite 5. Geneticists not invited to participate in further rounds 6. 18 completed round 1 but 1 was on maternity leave at the time of round 2 7. 36 completed round 1 but 2 were on mat leave at the time of round 2 8. Paediatricians combined with audiologists into group “audiologists/audiological physicians” 9. One participant had left the cleft service and retired since completing round 2 (11 or 12 completing round 2 invited). 10. One participant had left the cleft service since completing round 2 (8 of 9 completing round 2 invited) 11. One participant had left the cleft service since completing round 2 (13 of 14 completing round 2 invited) 12. One participant had left the cleft service since completing round 2 (27 of 28 completing round 2 invited) | | | | | | | | | |
