# Supplementary material for: The Importance of Integration of Stakeholder Views in Core Outcome Set Development: Otitis Media with Effusion in Children with Cleft Palate
Source: PLoS One. 2015 Jun 26;10(6):e0129514. doi: 10.1371/journal.pone.0129514 (PMC4483230; doi:10.1371/journal.pone.0129514)
Supplement: S5 Table — (DOCX) [file pone.0129514.s009.docx]

| **S5 Table. – summary of outcomes discussed at consensus meeting** | | | | | |
| --- | --- | --- | --- | --- | --- |
| Outcome | Number of the 8 stakeholder groups achieving consensus prior to meeting | Percentage of meeting participants scoring 7-9 | Percentage of meeting participants scoring 1-3 | Category of meeting conclusion   1. Discussed and voted 2. Discussed and agreed to combine with another outcome and to be considered as part of the “how” an outcome is measured 3. Discussed and agreed that further discussion with parents is needed 4. Agreed not to discuss further or vote. Not in the COS | Comments |
| Hearing | 8 | 100% | 0% | 1 |  |
| Chronic Otitis Media | 7 | 100% | 0% | 1 |  |
| Otitis media with effusion (OME) | 7 | 93% | 7% | 1 |  |
| Speech intelligibility | 6 | 85% | 0% | 2 | Also agreed by SAG post meeting to include as part of“how” speech development is measured. |
| Receptive language skills | 6 | 100% | 0% | 1 |  |
| Speech development | 6 | 93% | 7% | 1 |  |
| Atelectasis | 5 | 46% | 9% | 2 | Atelectasis to be combined with “Chronic Otitis Media” |
| Cholesteatoma | 5 | 84% | 0% | 3 |  |
| Psycho social development | 5 | 71% | 7% | 1 |  |
| Acute otitis media (AOM) | 5 | 78% | 7% | 1 |  |
| Consonant production | 5 | 76% | 8% | 2 | Also agreed by SAG post meeting to include as part of“how” speech development is measured.. |
| Necessity to remove ventilation tubes | 5 | 0% | 67% | 4 | Not in the COS as this relates to a specific treatment |
| Requirement for repeated ventilation tubes | 5 | 44% | 27% | 4 | Not in the COS as this relates to a specific treatment |
| Parental satisfaction with treatment | 5 | 69% | 8% | 1 |  |
| Child's satisfaction with treatment | 5 | 61% | 0% | 1 |  |
| Child's perspective of speech | 5 | 69% | 0% | 2 | Also agreed by SAG post meeting to include as part of“how” speech development is measured. |
| Persistent tympanic membrane retraction | 4 | N/A | N/A | 2 | Persistent tympanic membrane retraction to be combined with “Chronic Otitis Media” |
| Cognitive development | 4 | N/A | N/A | 2 | Cognitive development to be combined with “how well you are doing at school” |
| Developmental progress | 4 | N/A | N/A | 2 | Developmental progress to be combined with “how well you are doing at school” |
| Consonant production - cleft related speech patterns | 4 | N/A | N/A | 2 | Consonant production - cleft related speech patterns to be combined with “consonant production”  Also agreed by SAG post meeting to include as part of“how” speech development is measured. |
| Side effects of treatment | 4 | 100% | 0% | 1 |  |
| Listening skills * | 4 | 84% | 0% | 1 | Discussed and voted. Achieved “consensus in” but was not scored by parents or children therefore agreed to confirm inclusion in the COS with parents. |
| Psychosocial wellbeing * | 4 | 69% | 0% | 1 |  |
| Tympanosclerosis | 3 | N/A | N/A | 2 | Tympanosclerosis to be combined with “Chronic Otitis Media” |
| Persistent tympanic membrane perforation | 3 | N/A | N/A | 2 | Persistent tympanic membrane perforation to be combined with “Chronic Otitis Media” |
| Otalgia | 3 | 67% | 24% | 1 |  |
| Otorrhoea | 3 | 50% | 8% | 1 |  |
| Eustachian tube function | 3 | 27% | 0% | 3 |  |
| Expressive language skills | 3 | N/A | N/A | 3 | At the consensus meeting this was considered as part of the “how” speech development is measured and it was agreed not to vote. However, post meeting discussion with the SAG identified that the grouping of this outcome for parents might not have been appropriate and so this outcome should be discussed further. |
| Speech signs of velopharyngeal insufficiency | 3 | N/A | N/A | 3 | Wording of lay description should be revisited and discussed with parents |
| Early extrusion or blockage of ventilation tubes | 3 | N/A | N/A | 4 | Not in the COS as this relates to a specific treatment |
| Child stress | 3 | 51% | 26% | 1 |  |
| Psychological wellbeing* | 3 | N/A | N/A | 3 |  |
| Internalising behaviour | 2 | N/A | N/A | 4 |  |
| Externalising Behaviour | 2 | N/A | N/A | 4 |  |
| Academic achievement | 2 | 66% | 8% | 2 | Academic achievement to be combined with “how well you are doing at school” |
| Literacy | 2 | N/A | N/A | 2 | Literacy to be combined with “how well you are doing at school” |
| Phonological memory | 2 | N/A | N/A | 2 | To be combined with “how well you are doing at school”” |
| Tinnitus | 2 | 25% | 50% | 3 |  |
| Vertigo | 2 | 67% | 0% | 1 |  |
| Stapedial reflex | 2 | 0% | 50% | 1 |  |
| Parent's perspective of speech | 2 | N/A | N/A | 2 | Also agreed by SAG post meeting to include as part of“how” speech development is measured. |
| Parental stress | 2 | 43% | 14% | 1 |  |
| Intelligence | 1 | N/A | N/A | 2 | Intelligence to be combined with “how well you are doing at school” |
| Temporary tympanic membrane perforation | 1 | N/A | N/A | 4 |  |
| Upper Respiratory Tract Infection | 1 | 0% | 43% | 3 |  |
| Nasal obstruction | 0 | N/A | N/A | 4 |  |
| Rhinitis | 0 | N/A | N/A | 4 |  |
| Hyperacusis* | 0 | N/A | N/A | 3 |  |
| * not scored by parents and children therefore total number of stakeholder groups that could reach consensus = 7 | | | | | |
