# Supplementary material for: The Importance of Integration of Stakeholder Views in Core Outcome Set Development: Otitis Media with Effusion in Children with Cleft Palate
Source: PLoS One. 2015 Jun 26;10(6):e0129514. doi: 10.1371/journal.pone.0129514 (PMC4483230; doi:10.1371/journal.pone.0129514)
Supplement: S6 Table — (DOCX) [file pone.0129514.s010.docx]

| S5 Table: Stakeholder Representation at consensus meeting | | | | | |  |
| --- | --- | --- | --- | --- | --- | --- |
| Stakeholder Group | Number of voting members attending consensus meeting | Percentage Representation at consensus meeting | Number of voting members scoring eleven outcome at follow up meeting |  |  |  |
| ENT Surgeon | 2 | 14% | 0 |  |  |  |
| Cleft Nurse Specialist | 2 | 14% | 0 |  |  |  |
| Speech and Language Therapist | 4 | 29% | 0 |  |  |  |
| Audiologist/Audiological Physician | 2 | 14% | 0 |  |  |  |
| Cleft Surgeon | 1 | 7% | 1 |  |  |  |
| Clinical Psychologist | 0 | 0% | 0 |  |  |  |
| Parent/parent representative | 3 | 21% | 9 |  |  |  |
